# Identification of Urine Biomarkers to Improve Eligibility for Prostate Biopsy and Detect High-Grade Prostate Cancer

**DOI:** 10.3390/cancers14051135

**Published:** 2022-02-23

**Authors:** Nagjie Alijaj, Blaz Pavlovic, Paul Martel, Arnas Rakauskas, Valérie Cesson, Karim Saba, Thomas Hermanns, Pascal Oechslin, Markus Veit, Maurizio Provenzano, Jan H. Rüschoff, Muriel D. Brada, Niels J. Rupp, Cédric Poyet, Laurent Derré, Massimo Valerio, Irina Banzola, Daniel Eberli

**Affiliations:** 1 Department of Urology, University Hospital of Zürich and University of Zürich, 8006 Zürich, Switzerland; nagjielaila.alijaj@usz.ch (N.A.); blaz.pavlovic@usz.ch (B.P.); 2 Department of Urology, Urology Research Unit and Urology Biobank, University Hospital of Lausanne, 1011 Lausanne, Switzerland; paul.martel@chuv.ch (P.M.); arnas.rakauskas@chuv.ch (A.R.); valerie.cesson@chuv.ch (V.C.); laurent.derre@chuv.ch (L.D.); massimo.valerio@chuv.ch (M.V.); 3 Department of Urology, University Hospital of Zürich, 8091 Zürich, Switzerland; sabakarim@gmail.com (K.S.); thomas.hermanns@usz.ch (T.H.); pascal.oechslin@usz.ch (P.O.); markus.veit@usz.ch (M.V.); maurizio.provenzano@gmail.com (M.P.); cedric.poyet@usz.ch (C.P.); daniel.eberli@usz.ch (D.E.); 4 Department of Pathology and Molecular Pathology, University Hospital of Zürich, 8091 Zürich, Switzerland; janhendrik.rueschoff@usz.ch (J.H.R.); murieldiana.brada@usz.ch (M.D.B.); niels.rupp@usz.ch (N.J.R.); 5 Faculty of Medicine, University of Zürich, 8032 Zürich, Switzerland

**Keywords:** eligibility for prostate biopsy, prostate cancer, PSA, screening, urine biomarker

## Abstract

**Simple Summary:**

The screening of prostate cancer (PCa), based on the serum prostate specific antigen (PSA), is characterized by a high number of false positives, leading to overdiagnosis of healthy men and overtreatment of indolent PCa. This clinical problem severely affects the quality of life of patients, who would benefit from more specific risk stratification models. By performing a mass spectrometry (MS) screening on urine samples collected prior to prostate biopsy, we identified novel biomarkers and validated them by ELISA. Here, we show that an upfront urine test, based on quantitative biomarkers and patient age, has a higher performance compared to PSA (AUC = 0.6020) and is a feasible method to improve the eligibility criteria for prostate biopsy, to detect healthy men (AUC = 0.8196) and clinically significant PCa, thereby reducing the number of unnecessary prostate biopsies.

**Abstract:**

PCa screening is based on the measurements of the serum prostate specific antigen (PSA) to select men with higher risks for tumors and, thus, eligible for prostate biopsy. However, PSA testing has a low specificity, leading to unnecessary biopsies in 50–75% of cases. Therefore, more specific screening opportunities are needed to reduce the number of biopsies performed on healthy men and patients with indolent tumors. Urine samples from 45 patients with elevated PSA were collected prior to prostate biopsy, a mass spectrometry (MS) screening was performed to identify novel biomarkers and the best candidates were validated by ELISA. The urine quantification of PEDF, HPX, CD99, CANX, FCER2, HRNR, and KRT13 showed superior performance compared to PSA. Additionally, the combination of two biomarkers and patient age resulted in an AUC of 0.8196 (PSA = 0.6020) and 0.7801 (PSA = 0.5690) in detecting healthy men and high-grade PCa, respectively. In this study, we identified and validated novel urine biomarkers for the screening of PCa, showing that an upfront urine test, based on quantitative biomarkers and patient age, is a feasible method to reduce the number of unnecessary prostate biopsies and detect both healthy men and clinically significant PCa.

## 1. Introduction

Prostate cancer (PCa) is one of the most frequently diagnosed cancers worldwide and a prominent reason for tumor-related deaths in men [1]. In past years, early detection of PCa and its clinical management became a controversial topic. On the one hand, implementation of the serum biomarker prostate specific antigen (PSA), as a standard for the screening of PCa in the early 1990s, resulted in an increased diagnosis of early-stage tumors and a reduction of PCa-specific mortality rates [2]. Additional refinements in the PCa screening procedure due to new biomarkers and technologies, such as magnetic resonance imaging (MRI), have further improved the predictive performances of PSA [3]. On the other hand, specificities of current diagnostic examinations remain low and still lead to a high number of false positives, resulting in unnecessarily performed prostate biopsies [4]. Therefore, overdiagnosis of healthy men and overtreatment of indolent PCa remains a clinical challenge with significant impact on the quality of life of patients due to possible severe side effects [5,6]. To overcome this problem, more specific risk stratification models that can complement PSA testing need to be developed, to distinguish clinically significant from indolent PCa, and to reduce the number of biopsies performed.

Urine is an ideal clinical specimen for diagnostic testing. Its easy collection is completely non-invasive and it allows the processing of large volumes, compared to tissue, blood or other biological materials. This enables the detection of biomarkers at any time point during patient care and facilitates not only diagnosis, but also the monitoring of the disease. The detection of biomarkers in urine has been studied for a wide range of cancers with ultrasensitive screening methods, such as nuclear magnetic resonance (NMR) spectroscopy and mass spectrometry (MS) [7,8]. Specific metabolites were examined for their potential to screen for cancers of the urological system, but also for non-urological tumors such as lung, breast, colorectal, gastric, hepatic, pancreatic, and renal cancer [9].

The prostate epithelium secretes cellular substances into the gland and prostate cancer cells can be shed into the prostatic fluids, where they exude into the urine [10,11]. Sensitive assays can then detect DNA, RNA, proteins, and exosomes of tumor origin [12,13]. MS proteomics can be a powerful tool for high-throughput screening of proteins in urine and can be used for the identification of new biomarkers [14,15]. The translation of such methods into the clinic for standard diagnostic screening is elusive because of the high cost of instruments and the need for specially trained personnel. Therefore, validation studies of biomarkers are often performed on larger patient cohorts with immunological assays such as ELISA, which is a well-established method for protein quantification.

The aim of this study was to discover novel urine biomarkers for the detection of PCa and investigate their potential as an improved diagnostic test. The goal was to select, with high sensitivity, men with unspecifically elevated PSA from men who could benefit from prostate biopsy, which remains the standard of care for the diagnosis of PCa. Since low-grade PCas are generally considered indolent, the aim of the study was also to identify biomarkers for the selection of men harboring high-grade PCa. Thus, by improving the eligibility criteria for prostate biopsy, we would reduce the number of unnecessary prostate biopsies performed. Additionally, it might offer the possibility of non-invasive disease monitoring. Tests that rely on the quantification of single biomarkers are often limited in their power to predict cancer, a disease that is hallmarked by its heterogenic biology [16,17]. Therefore, we focused on the quantification of multiple biomarkers to achieve an increased accuracy in predicting PCa.

We performed a MS screening on urine samples from 45 men with elevated PSA levels scheduled for prostate biopsy and identified 2.735 proteins across all samples, as well as potential biomarkers for the detection of all grades of PCa or high-grade tumors only. Top candidates were then validated by ELISA and a combinatory analysis predicted their performances as multiplexed diagnostic test for PCa screening.

## 2. Materials and Methods

### 2.1. Urine Collection and Processing

A total of 45 patients were enrolled in the study at the Urology Department of the University Hospital of Zürich (Zürich, Switzerland). Samples were collected as first-morning urine from men not subjected to prostatic massage, with high serum PSA levels (≥2 ng/mL) and/or abnormal digital rectal examination (DRE) results, before the performance of the prostate biopsy. Sample aliquots were then stored at −80 °C until use. Patients’ recruitment, urine sample collection, and analysis were approved by the Ethics Committee of Kanton Zürich (BASEC n° 2016-00829).

### 2.2. Mass Spectrometry Analysis

Mass spectrometry (MS) analysis was performed by Biognosys AG (Schlieren, Switzerland). All solvents were HPLC-grade from Sigma Aldrich (Schaffhausen, Switzerland) and all chemicals, if not stated otherwise, were obtained from Sigma Aldrich (Schaffhausen, Switzerland).

#### 2.2.1. Sample Preparation

After thawing, sample digestion was performed on single filter units (Sartorius Vivacon 500, 30.000 MWCO HY) following a modified FASP protocol (described by the Max Planck Institute of Biochemistry, Martinsried, Germany). Samples were denatured with Biognosys’ Denature Buffer and reduced/alkylated using Biognosys’ Reduction/Alkylation Solution for 1 h at 37 °C. Subsequently, digestion to peptides was carried out using 1 μg trypsin (Promega) per sample, overnight at 37 °C.

#### 2.2.2. Clean-Up for Mass Spectrometry

Peptides were desalted using C18 Ultra Micro Spin columns (The Nest Group) according to the manufacturer’s instructions and dried down using a SpeedVac system. Peptides were resuspended in 17 μL LC solvent A (1% acetonitrile, 0.1% formic acid (FA)) and spiked with the Biognosys iRT kit calibration peptides. Peptide concentrations were determined using a UV/VIS Spectrometer (SPECTROstar Nano, BMG Labtech, Ortenberg, Germany).

#### 2.2.3. HPRP Fractionation

For HPRP fractionation of peptides, digested samples were pooled. Ammonium hydroxide was added to a pH value > 10. The fractionation was performed using a Dionex UltiMate 3000 RS pump (Thermo Scientific™) on an ACQUITY UPLC CSH C18 1.7 μm, 2.1 × 150 mm column (Waters). The gradient was 1% to 40% solvent B in 30 min, solvents were A: 20 mM ammonium formate in water, B: acetonitrile. Fractions were taken every 30 s and sequentially pooled to 12 fraction pools. These were dried down and resolved in 15 μL solvent A. Prior to mass spectrometric analyses, they were spiked with Biognosys’ iRT kit calibration peptides. Peptide concentrations were determined using a UV/VIS Spectrometer (SPECTROstar Nano, BMG Labtech).

#### 2.2.4. Shotgun LC–MS/MS for Spectral Library Generation

For shotgun LC–MS/MS measurements, 2 μg of peptides per fraction were injected to an in-house packed C18 column (Dr. Maisch ReproSil-Pur, 1.9 μm particle size, 120 Å pore size; 75 μm inner diameter, 50 cm length, New Objective) on a Thermo Scientific Easy nLC 1200 nano-liquid chromatography system connected to a Thermo Scientific™ Q Exactive™ HF mass spectrometer equipped with a standard nano-electrospray source. LC solvents were A: 1% acetonitrile in water with 0.1% FA; B: 15% water in acetonitrile with 0.1% FA. The nonlinear LC gradient was 1–52% solvent B in 60 min followed by 52–90% B in 10 s, 90% B for 10 min, 90–1% B in 10 s and 1% B for 5 min. A modified TOP15 method from Kelstrup was used [18]. Full MS covered the *m*/*z* range of 350–1650 with a resolution of 60.000 (AGC target value was 3 × 10^6^) and was followed by 15 data dependent MS2 scans with a resolution of 15.000 (AGC target value was 2 × 10^5^). MS2 acquisition precursor isolation width was 1.6 *m*/*z*, while normalized collision energy was centered at 27 (10% stepped collision energy) and the default charge state was 2+.

#### 2.2.5. HRM Mass Spectrometry Acquisition

For DIA LC–MS/MS measurements, 2 μg of peptides and 1 IE of PQ500 reference peptides were injected per sample. For samples with less than 2 μg of total peptide available, the amount of reference peptides was adjusted accordingly. Peptides were injected into an in-house packed C18 column (Dr. Maisch ReproSil-Pur, 1.9 μm particle size, 120 Å pore size; 75 μm inner diameter, 50 cm length, New Objective) on a Thermo Scientific Easy nLC 1200 nano liquid chromatography system connected to a Thermo Scientific Q Exactive HF mass spectrometer equipped with a standard nano-electrospray source. LC solvents were A: 1% acetonitrile in water with 0.1% FA; B: 15% water in acetonitrile with 0.1% FA. The nonlinear LC gradient was 1–55% solvent B in 120 min followed by 55–90% B in 10 s, 90% B for 10 min, 90–1% B in 10 s, and 1% B for 5 min. A DIA method with one full range survey scan and 22 DIA windows was used.

#### 2.2.6. Database Search of Shotgun LC–MS/MS Data and Spectral Library Generation

The shotgun mass spectrometric data were analyzed using Biognosys’ search engine SpectroMine™, the false discovery rate on peptide and protein level was set to 1%. A human UniProt FASTA database (Homo sapiens, accessed on 1 July 2019) was used for the search engine, allowing for two missed cleavages and variable modifications (N-term acetylation, methionine oxidation, deamidation (NQ), carbamylation (KR)). The results were used for generation of a sample-specific spectral library.

#### 2.2.7. HRM Data Analysis

HRM mass spectrometric data were analyzed using Spectronaut™ 14 software (Biognosys). The false discovery rate (FDR) on peptide and protein levels was set to 1% and data were filtered using row-based extraction. The spectral library generated in this study was used for the analysis. The HRM measurements analyzed with Spectronaut™ were normalized using global normalization.

#### 2.2.8. Data Analysis

For testing of differential protein abundance, MS1 and MS2 protein intensity information was used [19]. Protein intensities for each protein were analyzed using a two sample Student’s t-test, and *p*-values were corrected for overall FDR using the q-value approach [20]. The following thresholds were applied for candidate ranking: q-value < 0.05 and absolute average log2 ratio > 0.8074 (fold change > 1.75). After removal of proteins that were not identified in at least 90% of the samples, a selection based on ROC analysis was performed in order to identify the final list of the best performing 25 candidates (AUC > 0.670 and >10% specificity at 100% sensitivity).

### 2.3. ELISA Validation

Validation of mass spectrometry results was performed using commercially available ELISA kits and following the manufacturers’ protocols (Table S1). Before use, urine sample aliquots were equilibrated to room temperature. Measurements were conducted using the Epoch 2 microplate reader (BioTek, Zürich, Switzerland) and data were analyzed with the Gen5 software (version 2.09, BioTek, Zürich, Switzerland).

### 2.4. Immunohistochemical Staining of Prostate Tissues

For immunohistochemical evaluation a representative tissue block of *n* = 11 prostate adenocarcinoma cases, including periurethral tumor manifestations if available, was selected and stained for specific antibodies (Table S2). Staining and detection was performed using an automated staining system (Ventana). Semi-quantitative evaluation for each antibody was performed by two experienced pathologists. For each tissue block a corresponding hematoxylin–eosin (HE)-stained slide was available for morphological identification of prostate cancer. For each immunohistochemical marker the expression in the tumor and normal prostatic tissue were evaluated separately by assigning a four-tiered score (0 = negative, 1 = weak, 2 = moderate, 3 = strong). The extent of stained benign and malignant glands was estimated in 10% increments. In addition, the cellular compartment of the staining for both tumor area and normal prostatic glands was specified, whereas in the normal prostatic glands further evaluation of the distinct stained cell type (luminal and basal cells) was recorded. The predominant staining pattern was assessed when considerable heterogeneity of the staining intensity was detected.

### 2.5. Statistics and Data Analysis

All statistical analyses (except for mass spectrometry data) were performed with the GraphPad prism software, version 9. Continuous variables were expressed as box-plots (from the 25th to the 75th percentile and median), with whiskers representing the minimum and the maximum values. Statistical significance was calculated with the unpaired non-parametric Mann–Whitney U test.

For the characterization of single biomarkers, ROC curve analysis was performed applying the Wilson/Brown method, whereas for combinatorial analysis of non-correlated proteins, a multiple logistic regression was applied. The correlation matrix was assessed with the Pearson correlation method.

An online tool was used to draw volcano plots (VolcaNoseR, https://huygens.science.uva.nl/VolcaNoseR/, accessed on 8 September 2021).

## 3. Results

### 3.1. Patient Characteristics

A total of 45 consecutive men with suspected PCa were enrolled in this study and underwent a prostate biopsy after urine sample collection. Their demographic and clinical characteristics are summarized in Table 1, including age, serum PSA and prostate volume. Biopsy results are classified according to the Gleason score (GS) and evaluated for diagnostic purposes by genitourinary pathologists at the University Hospital of Zürich. PCa was detected in 46.7% (21/45) and clinically significant PCa (GS 7–9) in 37.8% of the patients. More precisely, 8.9% of the patients were diagnosed with GS 6, 17.8% with GS 7a/b, and 20.0% harbored a GS 8 or GS 9 tumor. Gleason score follow-up at repeated biopsies or upon prostatectomy showed that only one patient was upgraded.

**Figure 1:**
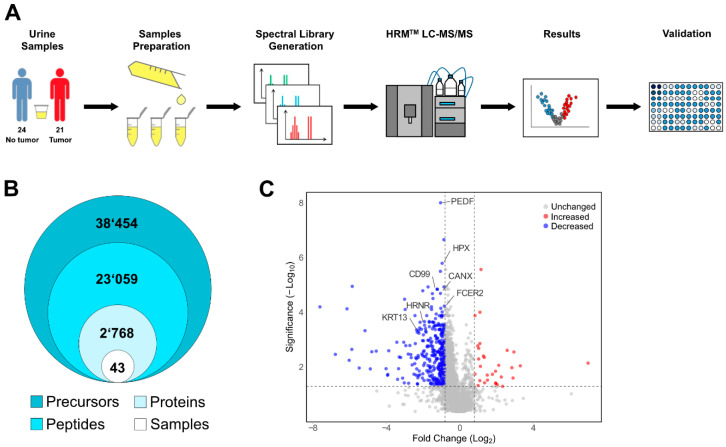
Identification of candidate urine biomarkers by mass spectrometry. (**A**) Schematic workflow overview of urine biomarker screening via mass spectrometry and validation with ELISA; (**B**) 2.768 proteins, 23.059 peptides, and 38.454 precursors were quantified across all 43 urine samples. (**C**) Volcano plot of 2.768 proteins quantified by mass spectrometry. The 351 differently distributed protein candidates are shown in blue (decreased in tumors) and red (increased in tumors) and were defined by: q-value < 0.05 and average fold change > 1.75. The seven candidates PEDF, HPX, CD99, CANX, FCER2, HRNR, and KRT13 are indicated.

Collected urine samples were then screened by MS and potential novel biomarkers analyzed by ELISA (Figure 1A).

### 3.2. Mass Spectrometry Screening and Selection of Urine Biomarkers for PCa Detection

For mass-spectrometry, a spectral peptide library was generated by shotgun LC–MS/MS of high-pH reversed-phase chromatography (HPRP) fractions from all 45 urine samples. Two samples showed a significant contamination with albumin, which led to the suppression of other peptide signals, and were therefore excluded from further analysis (data not shown). We identified a total of 38.454 precursors (peptides including different charges and modifications), corresponding to 23.059 unique peptides and 2.768 proteins across all 43 urine samples by using a false discovery rate of 1% (Figure 1B).

For the identification of candidate biomarkers to detect healthy men, we compared the abundance of 2.768 proteins in samples from patients not affected by tumor and those with PCa. Significantly dysregulated proteins were identified by setting the q-value below 0.05, at an average fold change of more than 1.75, resulting in 351 biomarker candidates (Figure 1C, Table S3). Strikingly, most of the candidates (321) displayed decreased levels in the urine of PCa patients compared to healthy men. In contrast, only 30 candidate biomarker candidates were found to have increased levels in the “tumor” group.

**Figure 2:**
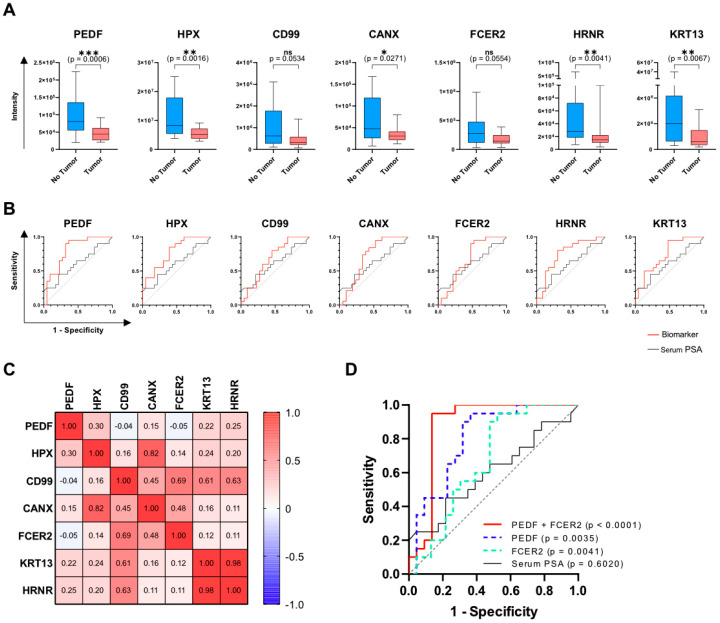
Potential candidate biomarkers for the detection of healthy men. Mass-spectrometry based quantification of the biomarkers (**A**) PEDF, HPX, CD99, CANX, FCER2, HRNR, and KRT13 in patients with and without PCa. Results are expressed as box-plots (from the 25th to the 75th percentile and median) with whiskers representing the minimum and the maximum values (Table S5). Statistical difference was assessed by the unpaired non-parametric Mann–Whitney U test with *p* ≤ 0.05 defined as statistically significant (ns *p* > 0.05; * *p* ≤ 0.05; ** *p* ≤ 0.01; *** *p* ≤ 0.001). (**B**) Diagnostic performances of the selected biomarkers assessed with the receiver operating characteristic (ROC). Each single biomarker (red curve) has a higher performance compared to serum PSA (black curve, AUC = 0.6020). (**C**) Correlation matrix assessed with the Pearson correlation method showing the correlation coefficients of the seven biomarkers with each other. A correlation between variables is defined as low for values up to ±0.3, medium for values up to ±0.5 and large for values up to ±1. (**D**) Combinatory analysis of non-correlating biomarkers via multiple logistic regression for the identification of tumor-free men. Coupling of PEDF and FCER2 resulted in the best performing biomarker combination, with an AUC of 0.8773 and a specificity of 72.7% at 100% sensitivity. Combined biomarkers displayed a higher performance compared to the single candidates and to serum PSA (black curve, AUC = 0.6020).

A key selection criterion for the best target molecules from the screening was the ability to discriminate healthy patients (with high specificity and accuracy), achieving a negligible number of false negatives (sensitivity > 90%). For this reason, all proteins that were not detected in more than three samples were excluded from further analysis. Additionally, proteins with low diagnostic performances, displaying a receiver operating characteristic (ROC) area under the curve (AUC) smaller than 0.670 and a specificity of less than 10% at 100% sensitivity, were removed. This ranking resulted in 43 biomarkers, with the top 25 candidates listed in Table S4. Among them, pigment epithelium-derived factor (PEDF), hemopexin (HPX), cluster of differentiation 99 (CD99), calnexin precursor (CANX), FCER2 (CD23, Fc fragment Of IgE receptor II), hornerin (HRNR), and keratin 13 (KRT13) showed remarkable diagnostic performance (Figure 2A,B; Table 2) and were selected for further validation by means of commercially available ELISA kits. Notably, all these biomarkers showed decreased levels in patients harboring prostate cancer.

The illustrated box plots in Figure 2A show the intensities of the biomarkers in patients with and without PCa as quantified by MS. All biomarkers identify true negative patients that could be spared from performing an unnecessary prostate biopsy, although the *p* value was a borderline result in terms of statistical significance for two biomarkers. The ROC plots (Figure 2B) show the ability of the single biomarkers to detect all PCa (GS 6–9, red curves) in comparison to the current standard of care, which is serum PSA (black curves). Each of the seven biomarkers had a superior performance compared to PSA and was able to correctly classify 100% of patients with PCa, while detecting tumor free men at varying specificities (Table 2).

Taken together, these data demonstrate that urine is a reliable proteomic source of biomarkers for the early detection of PCa and that the seven selected biomarker candidates are capable of sparing a relevant number of men from unnecessary prostate biopsy while avoiding misdiagnosis of patients bearing a prostate tumor.

### 3.3. Increase of PCa Detection Performance through Combinatory Analysis of Biomarkers

To assess potential biomarker combinations via multiple logistic regression, we first performed a Pearson correlation analysis among biomarker levels in the patient cohort (Figure 2C). In fact, the combination of variables can improve the performance of a predictive model only if the variables are not correlated to each other. In our analysis, we therefore combined biomarkers with a correlation coefficient of up to 0.3. Since the size of the cohort is limited to 43 patients, combinations of a maximum of two biomarkers were taken into consideration, in order to prevent the generation of overfitted models. All possible 14 combinations of biomarkers revealed a significantly larger AUC compared to the null hypothesis of AUC = 0.5 (Table 2). Moreover, any combination of two proteins led to a superior diagnostic performance, with increased AUC and higher specificity at 90% and 100% sensitivity compared to the single biomarkers. As an example, Figure 2D illustrates the multiple logistic regression curve of the PEDF and FCER2 combination (red line), which reached the best specificity of 72.7% at 100% sensitivity. This indicates that potentially 72.7% of healthy men could be spared from performing an unnecessary biopsy.

Our data show that the combination of biomarkers markedly improves the diagnostic power of the model and leads to the superior detection of healthy patients who could be spared from a prostate biopsy.

### 3.4. Validation of Biomarker Performance by ELISA

**Figure 3:**
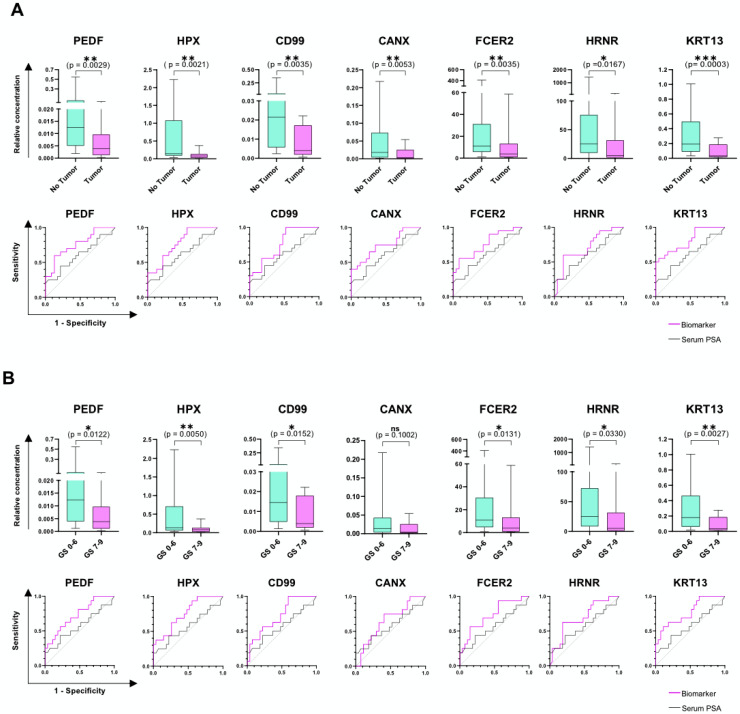
Validation of candidate biomarkers with ELISA for the detection of healthy men or high-grade PCa. Commercially available ELISA kits were used and results for PEDF, HPX, CD99, CANX, FCER2, HRNR, and KRT13 are represented as box-plots, where the relative concentration of the biomarkers normalized to two control molecules (CD44 and RNASE2) is compared for men with (**A**) no tumor to patients with any grade of PCa and (**B**) men with no tumor or low grade (GS = 6) PCa to patients harboring a high-grade tumor (GS ≥ 7). Significance was assessed with a statistical Mann–Whitney test (ns *p* > 0.05; * *p* ≤ 0.05; ** *p* ≤ 0.01; *** *p* ≤ 0.001). Results are expressed as box-plots (from the 25th to the 75th percentile and median) with whiskers representing the minimum and the maximum values (Table S5). The diagnostic potential of the single biomarkers was investigated with receiver operating characteristic (ROC) analysis. All biomarkers (purple curve) showed a better performance compared to serum PSA (black curve, all grades AUC = 0.6020; high-grade PCa AUC = 0.5690).

The validation of the candidate proteins selected from the MS analysis was performed by ELISA. Conversely to MS, immunoassays are standardized techniques that can be easily performed in any laboratory and allow for easy comparison among cohorts. For the MS measurements, the different urine samples were normalized according to their total peptide concentration and a defined amount of 2 µg was injected for each run. This approach cannot be applied to ELISA. Nevertheless, normalization is necessary to compensate for variations due to diet, time of collection and physiological characteristics of patients. Therefore, we have chosen non-dysregulated molecules from the mass-spectrometry analysis, i.e., cluster of differentiation 44 (CD44) and ribonuclease A family member 2 (RNASE2) and used them as controls for ELISA quantification of the single biomarkers (Figure S1; Table S4). Consistent with the corresponding MS data, Mann–Whitney U analysis of the normalized ELISA data for each analyte showed a significant difference between patients diagnosed with PCa and healthy individuals (Figure 3A). Furthermore, ROC curve analysis is concurrent with each MS dataset, demonstrating that all biomarkers have the diagnostic potential to detect healthy men at 100% sensitivity (Table 3).

Detection of high grade PCa has a relevant clinical impact, as it allows differentiation between patients who would benefit from active surveillance and those who need active treatments. We therefore also tested the potential of our biomarkers to discriminate also PCa GS ≥ 7. The quantitative analysis by ELISA shows that the seven biomarkers can detect high-grade PCa with high performance (Figure 3B, Table 3).

**Figure 4:**
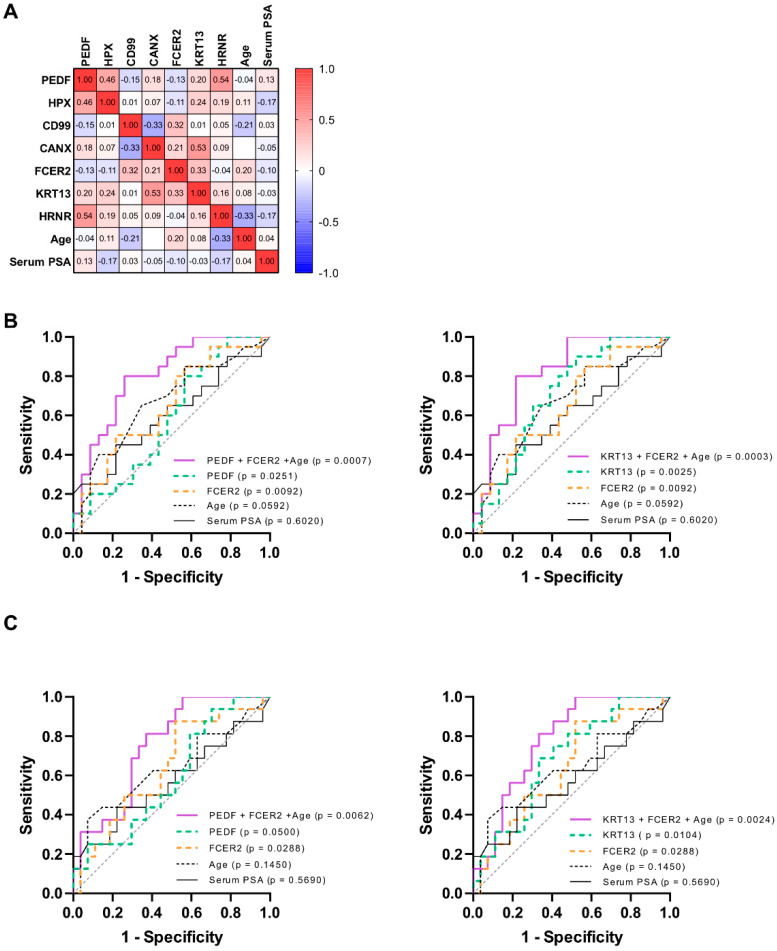
Multiple logistic regression analysis for the combination of biomarker levels (quantification by ELISA) with the patient’s age. (**A**) Pearson correlation matrix showing the correlation coefficients of the seven biomarkers, age and serum PSA with each other. A correlation between variables is defined as low for values up to ±0.3, medium for values up to ±0.5 and large for values up to ±1. (**B**) Combinatory analysis of immunoassay validation for the detection of healthy men. The combination of PEDF and FCER2 resulted as best pair from mass spectrometry and, in addition to age, achieved a final AUC of 0.8022 and a 39.1% specificity at 100% sensitivity. ELISA results revealed that, with an AUC of 0.8196 and a specificity of 52.2%, the best performing combination of biomarker was KRT13, FCER2, and age. Combined biomarkers showed a better performance compared to the single candidates and to serum PSA (black curve, AUC = 0.6020). (**C**) The combination of biomarkers with age can predict the presence of high-grade PCa. PEDF, FCER2, and age achieved a final AUC of 0.7523 and a 44.5% specificity at 100% sensitivity. By combining KRT13, FCER2, and age the performance reached an AUC of 0.7801 and a specificity of 48.1% (serum PSA is represented by the black curve, AUC = 0.5690).

When different biomarkers are normalized by the same controls, as in this study, their combinatory power is hampered by a highly correlated dataset (data not shown), driven by the identical normalization strategy. Hence, combinatorial analysis was performed by multiple logistic regression with non-normalized ELISA data. In this study, we excluded from the nomogram any clinical and demographic information with potentially high variability among individual clinics and cohorts. Prostate volume and digital rectal examination (DRE), for example, are known to be affected by the type of instrument used or by personnel expertise. We therefore included only the age of the patients as clinical variable to improve the predictive models. The Pearson correlation analysis of all variables is shown in Figure 4A. All combinations, including age, resulted in a significantly higher AUC compared to the null hypothesis and were able to detect all grades of PCa with 100% sensitivity (Table 4). As an example, the ROC curve of two of the best performing combinations, PEDF + FCER2 + age and KRT13 + FCER2 + age showed a specificity of 39.1% and 52.2% at 100% sensitivity, respectively (Figure 4B). Moreover, for the detection of high-grade tumors, the combination of uncorrelated analytes increased the overall performance of the single biomarkers. As model example, the ELISA quantification of KRT13, FCER2 + age showed a striking AUC of 0.7801 with a specificity of 48.1% at 100% sensitivity (Figure 4C).

Taken together, our data demonstrate that ELISA quantification of the biomarker candidates selected by MS is feasible and confirms the high diagnostic performance of the analytes, both as single and in combination for the detection of all PCa grades and clinically significant tumors (GS ≥ 7).

### 3.5. Immunohistochemical Analysis of Biomarker Expression in Malignant and Healthy Prostate Tissue

**Figure 5:**
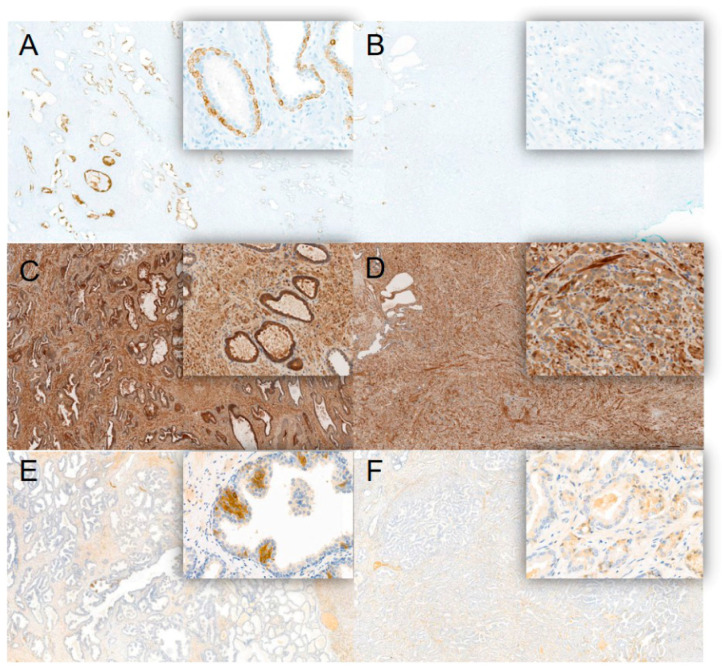
Immunohistochemical analysis of biomarker expression in benign and malignant prostate tissue. Overview (10× objective) of three biomarkers, which showed expression in basal cells, including respective magnifications (insets, 20× objective). (**A**) Positivity of KRT13 in basal cells of benign tissue, whereas (**B**) acinar adenocarcinoma shows loss of basal cells and KRT13 expression. (**C**) HPX showed in addition to expression in basal cells, reactivity in luminal cells of benign tissue, as well as obvious positivity in the fibromuscular stromal cells (background). (**D**) Prostate adenocarcinoma in comparison showed decreased expression of HPX. (**E**) PEDF showed reactivity in some of the basal cells, and weaker reactivity in luminal cells of the benign tissue. (**F**) In comparison, equally low expression in the (luminal) cells of the adenocarcinoma complexes.

To investigate the possible origin of the biomarkers, we performed immunohistochemistry analysis on prostate tissues from 11 men (of the initial 45 patients) that underwent radical prostatectomy. Because it was not possible to analyze prostate tissue from healthy patients, the healthy tissue areas of the prostate were used as control for each patient who underwent prostatectomy. The stainings were performed on tissue blocks, including benign and malignant areas of the prostate to compare biomarker expression levels. In concordance with the MS and ELISA data, KRT13 staining showed a distinct expression in benign and low expression in malignant tissue areas (Figure 5A,B; Table S6). We observed basal cell staining for KRT13, PEDF, and HPX in benign regions of the gland, a cell type that is absent in acinar-type adenocarcinomas (Figure 5A–F). Immunohistochemical analysis of CD99, HRNR, and CANX confirmed the expression of these markers in the prostate but, due to high heterogeneity, with high- and low-expression areas in both healthy and tumor tissues, it was not possible to compare the two conditions (Figure S2). No expression of the B-cell specific antigen FCER2 was detected in the prostate (Figure S2).

## 4. Discussion

Despite continuous improvements in the reduction of overdiagnosis and overtreatment of men suspected of having PCa, the number of healthy men that are subject to invasive procedures remains high [6,21]. This trend is concordant with our cohort. For this study, patients were selected for prostate biopsy only due to abnormal DRE results and/or elevated PSA levels. Approximately half (53.3%) of patients resulted having no tumor and should have been spared from performing the biopsy (Table 1).

Thus, the aim of this study was to identify novel urine biomarkers to improve the eligibility criteria for prostate biopsy and to more specifically discriminate PCa at an early stage, reducing the number of unnecessary biopsies. Here, we demonstrated the feasibility of diagnostic tests for the screening of PCa relying on urine biomarkers that can be routinely quantified by standardized laboratory methods such as ELISAs.

Urine samples were collected from patients before performing the biopsy and subjected to proteomic screening by mass-spectrometry (MS) to select biomarker candidates that are dysregulated when a prostate tumor is present. Although MS results showed promising results, the application of mass-spectrometry for urine analysis as routine diagnostic test is not feasible, due to the lack of a standard method to compare different batches of samples. A more practical approach is the implementation of quantitative immune-assays such as ELISA, which represents the gold standard for biomarker assessment and validation [22]. Consequently, among the 25 most performant candidates, seven proteins (PEDF, HPX, CD99, FCER2 (CD23), CANX, HRNR, and KRT13) were subsequently quantified in the same urine samples by quantitative ELISA. Additionally, their performance for the diagnosis of PCa and prediction of high-grade tumors was assessed. Although the translation of targeted MS assays into the clinical diagnostic setting appears to be difficult due to high costs and specific expertise requirements [23], the validation by ELISA demonstrates the feasibility of a clinical implementation through standard techniques. MS results of the 25 top ranked biomarkers in this study showed a significant decrease in signal intensity when a prostate tumor is present and can identify PCa patients with better performance compared to the standard PSA test (Table S4).

PEDF showed the best performance as a single biomarker, with AUC of 0.8023 and specificity of 36.4% at 100% sensitivity (Figure 2A,B). On the other hand, as an example of the many possible options (Figure 2D), the best performing combination of PEDF and FCER2 markedly increase the AUC in predicting PCa compared to each individual marker and also to PSA. Specifically, with this combination 72.7% of unnecessary biopsies could be avoided, without missing any patient with PCa (100% sensitivity).

The proteomic content of urine is affected by many factors, such as individual life-style, diet and time of sampling. For this reason, absolute biomarker data need to be normalized with a different strategy compared to MS, in which normalization is based on the overall cohort protein content. Figure 3A shows normalized ELISA results of the biomarkers panel, where each single molecule shows a strong diagnostic performance, in concurrence with the MS data. By combining KRT13 and FCER2 with age, we reached an AUC of 0.8196 and a specificity 52.2% at 100% sensitivity (Figure 4B). Besides the early detection of PCa, risk stratification of patients to better select clinically significant tumors is important to support optimal treatment options. For this reason, we have assessed the ability of the seven biomarkers to also detect tumors with GS ≥ 7 as well. Figure 3B shows that all candidates can predict the presence of high-grade PCa more precisely than serum PSA. The combination of KRT13 and FCER2 with age for the detection of high-grade PCa reached an AUC of 0.7801 and a specificity of 48.1% at 100% sensitivity (Figure 4C), thus potentially reducing the number of unnecessary biopsies almost by half, without missing any patient with clinically relevant PCa. Depending on the clinic, region and patients’ characteristics (e.g., age and expectation of life), men with low grade PCa (GS 6) will either be monitored or treated by local therapy options. In both cases, the novel biomarker panel can be applied to reduce unnecessary biopsies and monitor patients continuously and non-invasively. Therefore, by combining different biomarkers, we observed a relevant reduction of unnecessary biopsies, either performed on healthy individuals or on patients affected by clinically indolent tumors.

A relevant portion of the proteins identified in our study has already been described in other mass-spectrometry analyses of urine and to a lesser extent, in urinary extracellular vesicles, plasma or prostate tissue of patients. The seven biomarkers validated in our study were chosen exclusively based on their ability to predict PCa prior to biopsy and not considering their biological function. Nevertheless, some of them have been reported to be related to cancer. Although signal reduction in case of tumor progression as described for the seven biomarkers might be surprising, both literature and tissue analysis performed in this study support these findings. Hornerin (HRNR), a member of the fused-type S100 protein family, was shown to be expressed and to play a role in different tumor types [24,25,26]. Other members of the same protein family were examined in prostate tissue of PCa patients, demonstrating that the loss of S100A2 and increased expression of S100A4 are hallmarks of PCa progression [27]. Similarly, the prostate tissue analysis of the pigment epithelium-derived factor (PEDF), a natural angiogenesis inhibitor in prostate and pancreas [28,29], showed minimal expression in high grade PCa (GS 7–10), in contrast to healthy prostate tissue, where the staining shows high intensity [28]. The downregulation of CD99 was already shown to be essential for tumorigenesis. This has been described for several tumors [30,31,32], including prostate cancer [33]. In fact, the overexpression of CD99 in prostate cancer cells inhibited their migration and metastatic potential in both in vitro and in vivo experiments [31]. Hemopexin (HPX) has been described to be downregulated in urine from PCa patients compared to tumor free men, an observation that is in concordance with our findings [34]. Moreover, a bioinformatics analysis of multiple urinary and tissue proteomes revealed HPX downregulation in high-grade PCa compared to healthy tissue [35]. In contrast to our results, elevated levels in cancer have been reported for the remaining molecules. Increased levels of the Fc fragment of IgE receptor II (FCER2) have been implicated in different hematological malignancies and sarcomas [36,37,38,39,40,41]. In addition, FCER2 is expressed in subsets of B cells and in particular depicts follicular dendritic cell networks [42], whereas expression changes in urine could reflect an altered immune microenvironment in prostate adenocarcinoma patients. Keratin 13 (KRT13) belongs to the type I keratin family and its reduced expression has been associated with oral squamous cell carcinoma lesions [43,44,45] and bladder cancer [46]. In contrast to our results, a study in 2016 revealed a correlation between KRT13 tissue expression and prostate cancer metastasis [47]. However, as we could show expression of KRT13 in the basal cells of benign glands, and since the loss of basal cells is one hallmark of prostate adenocarcinoma [48], lower expression levels in urine could also be explained by increased tumoral occupation of the gland. The endoplasmic reticulum chaperone calnexin (CANX) is associated with newly synthesized glycoproteins and involved in correct protein folding [49]. So far, CANX has not been described in PCa but its altered expression has been associated with other cancers [50,51]. To the best of our knowledge, this is the first study to suggest a putative role in PCa for the above-described biomarkers in PCa, demonstrating their dysregulation at such an early stage (prior to biopsy) and the feasibility of their quantitative assessment in urine.

To investigate the possible origin of the biomarkers and their route to the urine, we performed a sequence-based analysis, predicting secretion pathways of proteins with the SecretomeP 2.0 server (http://www.cbs.dtu.dk/services/SecretomeP/, accessed on 5 October 2021). PEDF, HPX, CD99, and CANX are expressed with signal peptides and potentially traffic through the classical pathway (Golgi apparatus), whereas membrane protein FCER2 was predicted to traffic through a non-classical pathway. Conversely, KRT13 and HRNR do not appear to be secreted. This suggests that the proteins detected may be present in urine due to either the presence of cellular debris or particles deriving directly from the prostate or through blood filtration.

The prostate tissue analysis performed in this study confirms that six out of seven biomarkers validated by ELISA are expressed in prostatic adenocarcinomas. Intensity analysis shows that KRT13 levels are lower in tumor tissue compared to healthy prostate, in agreement with the MS and ELISA data. Tissue staining further revealed that KRT13, PEDF, and HPX are predominantly expressed in basal cells of the benign tissue, whereas they are not detected in tumor areas where basal cells have been lost. Notably, these findings are in support of the decreased levels detected in urine of PCa patients, as the basal cells might be responsible for the direct shedding or secretion of these biomarkers into the acinar lumen and thus the loss of expression of the biomarkers can be reflected in their dysregulated levels detected in the cohort. The heterogeneous expression of CD99, HRNR, and CANX in both healthy and tumor tissue hampered the quantitative comparison. FCER2 was not detected in prostate tissue and might derive from immune cells, as it is known to be expressed in B lymphocytes [52], thus suggesting that a relevant involvement of the immune system in PCa could be detected in urine at an early stage.

The present study has some limitations. First, it is a retrospective and single institution based study. Second, it relies on a small sample size, combining data of 43 patients for biomarker identification and validation. This became particularly evident when performing the multiple logistic regression analysis, as the cohort size determines the number of variables that can be combined to improve the model. To avoid false associations and large standard errors, a minimum number of five to ten events per predictor variable (EPV) has to be considered [53]. Since our cohort comprises 23 healthy men, we included no more than two to four predictor variables. Future studies investigating larger cohort sizes will allow the inclusion of higher numbers of variables and thereby improve their diagnostic performance. Nevertheless, for an explorative analysis of the biomarker candidates, the cohort provided a sufficient sample size and the combination of two to three variables yielded robust prediction models. Although it was currently not possible to validate the biomarkers in an independent cohort, their performance in this study was proved by use of two different and independent quantitative technologies, and the concordance of the findings underscores the importance of further validation of the targets.

## 5. Conclusions

In conclusion, here, we demonstrated that an upfront urine test based solely on the quantification of novel biomarkers is a feasible approach to improve eligibility criteria for a prostate biopsy and to detect the presence of high-grade PCa, independent of serum PSA, digital rectal examination, and clinical variables. The clinical implementation of a simple urine test represents one possible and safe way to reduce the overdiagnosis and overtreatment of PCa. Furthermore, since it is completely non-invasive, it could potentially be used for disease monitoring and active surveillance.

## 6. Patents

This study was submitted for patent application (applicant: University of Zürich; inventors: I. Banzola, N. Alijaj, B. Pavlovic, D. Eberli). The patent application was submitted to the European patent office (application number: EP 21/215742.4).

## Figures and Tables

**Table 1:** Characteristics of the patients. Statistical analysis was performed using a Mann–Whitney U test, which showed age as the only variable significantly different between the tumor vs. non-tumor patients (age: *p* = 0.048; PSA: *p* = 0.323; prostate volume: *p* = 0.164). * Data available for only 41 patients.

	Number of Samples (% of Total)	Gleason Score	Median Age (Min–Max)	Median Serum PSA (Min–Max)	Prostate Volume (Min–Max) *
No Tumor	24 (53.3%)	0	63.5 (52–82)	6.60 (2.00–14.97)	60.19 (18.56–203.68)
Tumor	21 (46.7%)	6–9	65 (52–76)	7.22 (2.00–38.80)	48.59 (17.00–80.63)
	4 (8.9%)	6	65 (64–70)	8.53 (4.53–17.37)	60.54 (30.90–80.63)
	8 (17.8%)	7	65 (52–73)	4.94 (2.00–11.00)	50.00 (26.45–72.54)
	9 (20.0%)	8–9	74 (58–76)	12.41 (4.86–38.80)	47.17 (17.00–60.00)
Total	45 (100%)		65 (52–82)	6.90 (2.00–38.80)	52.00 (17.00–203.68)

**Table 2:** ROC curve and multiple logistic regression analysis of the mass spectrometry results. The analysis was performed on the seven biomarker candidates and their possible non-correlating combinations for the identification of healthy men.

Biomarker	AUC	Std. Error	95% Confidence Interval	*p*-Value	Specificity at 90% Sensitivity	Specificity at 100% Sensitivity
PEDF	0.8023	0.070	0.6659 to 0.9386	0.0008	68.2	36.4
HPX	0.7761	0.070	0.6396 to 0.9125	0.0020	52.2	39.1
HRNR	0.7522	0.076	0.6033 to 0.9010	0.0047	47.8	13.0
KRT13	0.7391	0.075	0.5913 to 0.8869	0.0074	52.2	30.4
CANX	0.7043	0.085	0.5377 to 0.8708	0.0273	47.6	38.1
CD99	0.6750	0.083	0.5114 to 0.8386	0.0525	36.4	31.8
FCER2	0.6717	0.084	0.5075 to 0.8360	0.0544	52.2	30.4
PEDF + HPX	0.8977	0.050	0.7999 to 0.9956	<0.0001	72.7	50.0
PEDF + CD99	0.8786	0.056	0.7689 to 0.9883	<0.0001	76.2	66.7
PEDF + FCER2	0.8773	0.063	0.7530 to 1.000	<0.0001	86.4	72.7
PEDF + KRT13	0.8705	0.055	0.7618 to 0.9791	<0.0001	72.7	54.5
PEDF + HRNR	0.8568	0.058	0.7437 to 0.9699	<0.0001	77.3	54.5
PEDF + CANX	0.9105	0.053	0.8067 to 1.000	<0.0001	85.0	70.0
HPX + HRNR	0.8739	0.054	0.7682 to 0.9797	<0.0001	73.9	34.8
HPX + KRT13	0.8413	0.061	0.7211 to 0.9615	0.0001	60.9	56.5
HRNR + CANX	0.8496	0.062	0.7272 to 0.9720	0.0002	66.7	66.7
HPX + FCER2	0.8000	0.068	0.6670 to 0.9330	0.0008	60.9	60.9
HPX + CD99	0.7864	0.071	0.6462 to 0.9265	0.0015	63.6	54.5
KRT13 + CANX	0.7820	0.076	0.6322 to 0.9318	0.0023	61.9	61.9
KRT13 + FCER2	0.7652	0.074	0.6193 to 0.9111	0.0030	60.9	47.8
HRNR + FCER2	0.7457	0.076	0.5964 to 0.8949	0.0059	60.9	34.8

**Table 3:** ROC analysis of the ELISA results for the detection of healthy men and high-grade PCa. The table shows the diagnostic performance of ELISA results obtained normalizing the concentration of the seven candidates with two control molecules (CD44 and RNASE2). The “all PCa grades” analysis identifies healthy men (reaching 100% sensitivity at a specific threshold), whereas the “high-grade (GS 7–9) PCa” analysis identifies true negatives as either healthy men or patients harboring GS 6 PCa (reaching 100% sensitivity at a specific threshold).

	Biomarker	AUC	Std. Error	95% Confidence Interval	*p*-Value	Specificity at 90% Sensitivity	Specificity at 100% Sensitivity
**All PCa grades**	KRT13	0.8087	0.066	0.6797 to 0.9377	0.0005	43.5	43.5
	HPX	0.7696	0.071	0.6314 to 0.9077	0.0025	47.8	43.5
	PEDF	0.7609	0.073	0.6176 to 0.9041	0.0035	34.8	30.4
	CD99	0.7565	0.073	0.6136 to 0.8994	0.0041	52.2	47.8
	FCER2	0.7565	0.074	0.6114 to 0.9017	0.0041	47.8	13.0
	CANX	0.7457	0.076	0.5971 to 0.8942	0.0059	30.4	26.1
	HRNR	0.7120	0.080	0.5553 to 0.8686	0.0176	39.1	17.4
**High-grade PCa**	KRT13	0.7708	0.075	0.6247 to 0.9170	0.0033	40.7	37.1
	HPX	0.7546	0.074	0.6094 to 0.8998	0.0057	44.4	37.0
	PEDF	0.7292	0.079	0.5752 to 0.8831	0.0129	33.3	29.6
	FCER2	0.7269	0.081	0.5690 to 0.8847	0.0138	44.4	11.2
	CD99	0.7222	0.078	0.5688 to 0.8756	0.0159	40.7	40.7
	HRNR	0.6956	0.083	0.5321 to 0.8591	0.0337	37.0	14.8
	CANX	0.6528	0.086	0.4849 to 0.8207	0.0973	25.9	22.1

**Table 4:** ROC curve and multiple logistic regression analysis of the ELISA results for the detection of healthy men or high-grade PCa. The seven single biomarkers (not normalized) and their combinations (including patients’ age as variable) were analyzed. The “all PCa grades” analysis identifies healthy men (reaching 100% sensitivity at a specific threshold), whereas the “high-grade (GS 7–9) PCa” analysis identifies true negatives as either healthy men or patients harboring GS 6 PCa (reaching 100% sensitivity at a specific threshold).

	Biomarker	AUC	Std. Error	95% Confidence Interval	*p*-Value	Specificity at 90% Sensitivity	Specificity at 100% Sensitivity
**All PCa grades**	KRT13	0.7696	0.071	0.6298 to 0.9093	0.0025	52.2	30.4
	HRNR	0.7413	0.079	0.5865 to 0.8961	0.0069	52.2	8.7
	FCER2	0.7326	0.077	0.5813 to 0.8839	0.0092	52.2	39.1
	CANX	0.7043	0.080	0.5479 to 0.8608	0.0221	30.4	17.4
	PEDF	0.700	0.081	0.5404 to 0.8596	0.0251	30.4	30.4
	HPX	0.6978	0.081	0.5386 to 0.8570	0.0267	39.1	8.7
	CD99	0.6652	0.083	0.5032 to 0.8273	0.0642	34.8	21.7
	KRT13 + FCER2	0.8196	0.065	0.6927 to 0.9464	0.0003	52.2	52.2
	HPX + FCER2	0.8087	0.067	0.6767 to 0.9407	0.0005	43.5	30.4
	PEDF + FCER2	0.8022	0.067	0.6714 to 0.9329	0.0007	52.2	39.1
	HPX + KRT13	0.7826	0.070	0.6462 to 0.9190	0.0015	52.2	30.4
	HRNR + FCER2	0.7826	0.071	0.6429 to 0.9223	0.0015	56.5	13.0
	PEDF + KRT13	0.7804	0.070	0.6431 to 0.9178	0.0017	52.2	39.1
	KRT13 + CANX	0.7609	0.072	0.6189 to 0.9028	0.0035	47.8	30.4
	HPX + HRNR	0.7478	0.078	0.5960 to 0.8997	0.0055	43.5	8.7
	PEDF + CANX	0.7348	0.077	0.5844 to 0.8852	0.0085	47.8	26.1
	HRNR + CANX	0.7326	0.079	0.5781 to 0.8871	0.0092	43.5	8.7
	PEDF + CD99	0.7304	0.076	0.5808 to 0.8801	0.0099	43.5	34.8
	PEDF + HRNR	0.7283	0.080	0.5723 to 0.8842	0.0106	43.5	8.7
	HPX + CD99	0.7283	0.078	0.5753 to 0.8812	0.0106	39.1	17.4
	PEDF + HPX	0.7000	0.081	0.5417 to 0.8583	0.0251	26.1	13.0
**High-grade PCa**	KRT13	0.7361	0.077	0.5854 to 0.8868	0.0104	40.7	25.9
	HRNR	0.7199	0.084	0.5551 to 0.8847	0.0170	14.8	7.4
	FCER2	0.7014	0.079	0.5468 to 0.8560	0.0288	44.4	33.3
	HPX	0.6968	0.087	0.5262 to 0.8673	0.0327	7.4	7.4
	PEDF	0.6806	0.085	0.5141 to 0.8470	0.0500	33.3	18.5
	CD99	0.6644	0.086	0.4967 to 0.8320	0.0744	29.6	18.5
	CANX	0.6574	0.085	0.4907 to 0.8241	0.0875	22.2	14.8
	HPX + FCER2	0.7894	0.077	0.6376 to 0.9411	0.0017	33.3	33.3
	HPX + KRT13	0.7870	0.073	0.6432 to 0.9308	0.0018	33.3	18.5
	KRT13 + FCER2	0.7801	0.069	0.6447 to 0.9155	0.0024	51.8	48.1
	HPX + CD99	0.7662	0.078	0.6136 to 0.9188	0.0039	29.6	14.8
	PEDF + FCER2	0.7523	0.073	0.6090 to 0.8956	0.0062	48.1	44.5
	HRNR + FCER2	0.7523	0.076	0.6024 to 0.9022	0.0062	51.8	11.1
	HPX + HRNR	0.7500	0.084	0.5845 to 0.9155	0.0067	11.1	7.4
	PEDF + KRT13	0.7431	0.075	0.5964 to 0.8898	0.0083	44.5	33.3
	KRT13 + CANX	0.7384	0.076	0.5886 to 0.8882	0.0097	40.7	29.6
	PEDF + CD99	0.7176	0.078	0.5657 to 0.8695	0.0182	37.0	37.0
	PEDF + HPX	0.7083	0.083	0.5461 to 0.8705	0.0237	14.8	14.8
	HRNR + CANX	0.7014	0.083	0.5384 to 0.8644	0.0288	29.6	3.7
	PEDF + HRNR	0.6968	0.082	0.5358 to 0.8577	0.0327	33.3	11.1
	PEDF + CANX	0.6898	0.081	0.5303 to 0.8493	0.0394	44.4	18.5

## Data Availability

All data presented in this study are available in the manuscript and in the supplementary materials. Additional information are available for bona fide researchers who request it from the authors.

## Supplementary Materials

The following supporting information can be downloaded at: https://www.mdpi.com/article/10.3390/cancers14051135/s1, Figure S1: Mass spectrometry analysis of two possible control molecules; Figure S2: Representative images of HRNR, CD99, CANX and FCER2 immunohistochemical stainings in one prostate adenocarcinoma patient (10× objective); Table S1: Commercial ELISA kits used for the validation of biomarker candidates; Table S2: Antibodies used for the immunohistochemical staining of prostate tissues; Table S3: Ranked candidate biomarkers from the MS screening for the detection of PCa; Table S4: Top 25 biomarkers and two control molecules resulted from mass spectrometry screening; Table S5: Statistical analysis of the biomarkers’ mass-spectrometry and ELISA quantification results; Table S6: Immunohistochemical staining of eleven prostate adenocarcinoma cases.

## Abbreviations

PCa	prostate cancer
PSA	prostate specific antigen
MS	mass spectrometry
AUC	area under the curve
MRI	magnetic resonance imaging
NMR	nuclear magnetic resonance
ELISA	enzyme-linked immunosorbent assay
DRE	digital rectal examination
HPLC	high-performance liquid chromatography
FA	formic acid
UV/VIS	ultraviolet–visible
HPRP	high-pH reversed-phase chromatography
LC–MS	liquid chromatography–mass spectrometry
HRM	high resolution mass spectrometry
DIA	data-independent acquisition
FDR	false discovery rate
ROC	receiver operating characteristic
HE	Hematoxylin–eosin
GS	Gleason score
EPV	events per predictor variable
PEDF	pigment epithelium-derived factor
HPX	hemopexin
CD99	cluster of differentiation 99
CANX	calnexin precursor
FCER2	Fc fragment Of IgE receptor II
HRNR	hornerin
KRT13	keratin 13
CD44	cluster of differentiation 44
RNASE2	ribonuclease A family member 2
