# Supplementary material for: Identification of Urine Biomarkers to Improve Eligibility for Prostate Biopsy and Detect High-Grade Prostate Cancer
Source: Cancers (Basel). 2022 Feb 23;14(5):1135. doi: 10.3390/cancers14051135 (PMC8909910; doi:10.3390/cancers14051135)
Supplement: Supplementary file 1 [file cancers-14-01135-s001.zip › Supplementary Figures.pdf]

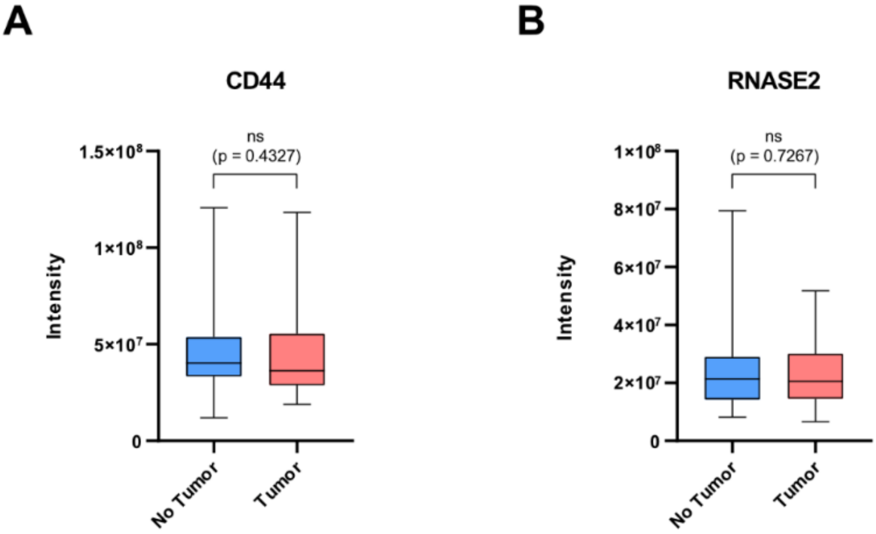

Figure S1: Mass spectrometry analysis of two possible control molecules. Mass spectrometry analysis of two control molecules. Mass-spectrometry quantification of CD44 (**A**) and RNASE2 (**B**) showed no significant difference in healthy men compared to patients with PCa, making both molecules good candidates as ELISA data normalizers. A Mann-Whitney test was performed to determine significance.

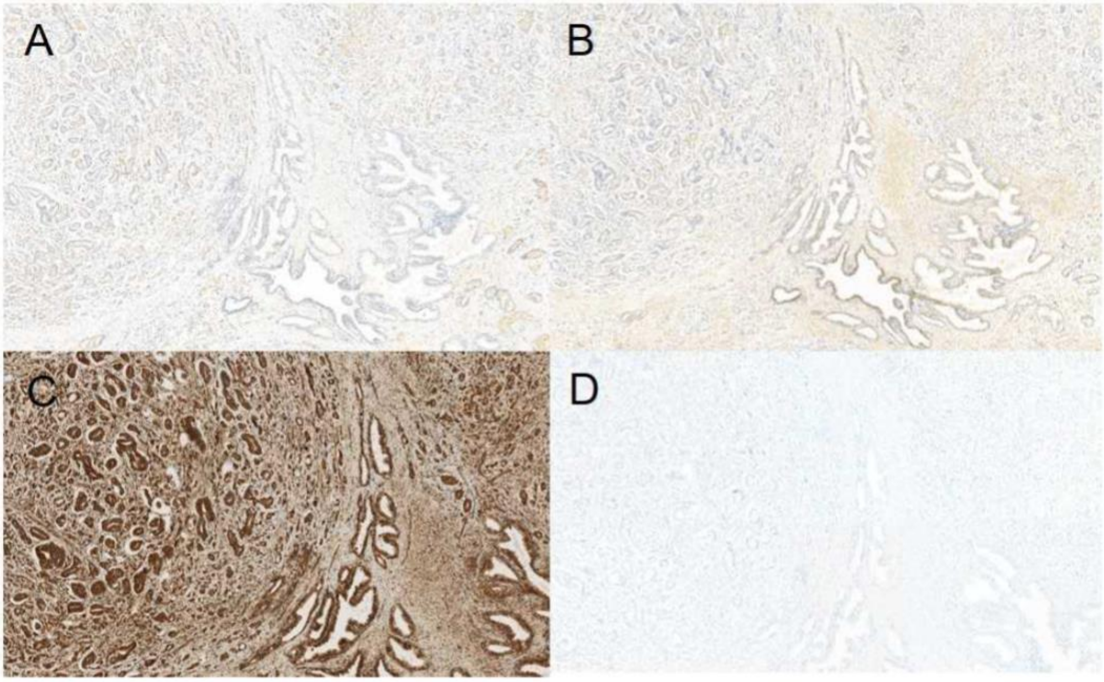

Figure S2: Representative images of HRNR, CD99, CANX and FCER2 immunohistochemical stainings in one prostate adenocarcinoma patient (10x objective). (**A**,**B**) showing low expression of HRNR and CD99, respectively, whereas (**C**) shows obvious and diffuse staining of CANX in benign and malignant tissue, as well as fibromuscular stroma. (**D**) There was no expression of FCER2 in any type of prostatic tissue.
