# Supplementary material for: Identification of Urine Biomarkers to Improve Eligibility for Prostate Biopsy and Detect High-Grade Prostate Cancer
Source: Cancers (Basel). 2022 Feb 23;14(5):1135. doi: 10.3390/cancers14051135 (PMC8909910; doi:10.3390/cancers14051135)
Supplement: Supplementary file 1 [file cancers-14-01135-s001.zip › Supplementary Tables.pdf]

Table S1: Commercial ELISA kits used for the validation of biomarker candidates.

Target	ELISA Catalogue Number	ELISA Producer	Notes
CD99	ELH-CD99	RayBiotech (Peachtree Corners, GA, USA)	-
HRNR	LS-F8355	LifeSpan BioScience (Seattle, WA, USA)	-
KRT13	LS-F36678	LifeSpan BioScience (Seattle, WA, USA)	-
FCER2	LS-F2751	LifeSpan BioScience (Seattle, WA, USA)	-
PEDF	LS-F33276	LifeSpan BioScience (Seattle, WA, USA)	-
HPX	EH238RB	Thermo Fisher Scientific (Basel, Switzerland)	-
CANX	ABIN6965340	Antibodies Online (Aachen, Germany)	-
CD44	ab45912	Abcam (Cambridge, UK)	Samples diluted 1:100/200
RNASE2	7630	MBL Int. Corp. (Woburn, MA, USA)	Samples diluted 1:50

Table S2: Antibodies used for the immunohistochemical staining of prostate tissues.

Target	Antibody clone	Antibody Producer	Dilution
CD99	HO36-1.1	Novocastra Laboratories	1:25
HRNR	polyclonal	Sigma Aldrich	1:500
KRT13	KS-1A3	Novocastra Laboratories	1:500
FCER2	1B12	Novocastra Laboratories	1:20
PEDF	polyclonal	Sigma Aldrich	1:50
HPX	EPR5610	Abcam	1:500
CANX	polyclonal	Invitrogen	1:500

Table S3: Ranked candidate biomarkers from the MS screening for the detection of PCa. The ranking is based on thresholds for q-values and fold change, resulting in 351 protein candidates.

Accession ID	Genes	Condition Numerator	Condition Denominator	AVG Log2 Ratio	Absolute AVG Log2 Ratio	Pvalue	Qvalue	% Change	Ratio
P36955	SERPINF1	6+7+8+9	0	-1.03931989	1.03931989	8.60E-12	9.61E-09	-51.344321	0.48655679
P27797	CALR	6+7+8+9	0	-0.86009261	0.86009261	3.86E-10	2.16E-07	-44.9082809	0.55091719
P02790	HPX	6+7+8+9	0	-0.95244333	0.95244333	4.25E-09	1.58E-06	-48.3243453	0.51675655
P62857	RPS28	6+7+8+9	0	1.16094985	1.16094985	9.74E-09	2.65E-06	123.604597	2.23604597
P33151	CDH5	6+7+8+9	0	-1.04928923	1.04928923	1.66E-08	3.10E-06	-51.6793835	0.48320617
P00491	PNP	6+7+8+9	0	-5.84523507	5.84523507	6.87E-08	1.10E-05	-98.2605622	0.01739438
P25787	PSMA2	6+7+8+9	0	-1.71925686	1.71925686	9.90E-08	1.15E-05	-69.6294879	0.30370512
P06727	APOA4	6+7+8+9	0	-0.85483935	0.85483935	1.03E-07	1.15E-05	-44.7073106	0.5529269
P14209	CD99	6+7+8+9	0	-1.23124337	1.23124337	1.66E-07	1.40E-05	-57.4049814	0.42595019
P02647	APOA1	6+7+8+9	0	-1.21375838	1.21375838	1.72E-07	1.40E-05	-56.8856026	0.43114397
P68871	HBB	6+7+8+9	0	-2.0069697	2.0069697	2.14E-07	1.59E-05	-75.1204844	0.24879516
P27824	CANX	6+7+8+9	0	-1.04504889	1.04504889	2.84E-07	1.99E-05	-51.5371514	0.48462849
Q8IX30	SCUBE3	6+7+8+9	0	-1.48501093	1.48501093	3.09E-07	2.03E-05	-64.2754171	0.35724583
P32241	VIPR1	6+7+8+9	0	-1.45214553	1.45214553	5.21E-07	3.06E-05	-63.4522507	0.36547749
P02812;P04280	PRB2;PRB1	6+7+8+9	0	-2.99633019	2.99633019	5.83E-07	3.26E-05	-87.468163	0.12531837
P06734	FCER2	6+7+8+9	0	-0.8338376	0.8338376	1.14E-06	5.78E-05	-43.896511	0.56103489
Q14515	SPARCL1	6+7+8+9	0	-1.53270244	1.53270244	1.32E-06	6.14E-05	-65.4370669	0.34562933
Q9NZT2	OGFR	6+7+8+9	0	-7.60567836	7.60567836	1.38E-06	6.18E-05	-99.486592	0.00513408
P08185	SERPINA6	6+7+8+9	0	-1.02690793	1.02690793	1.68E-06	6.97E-05	-50.9239149	0.49076085
O75110	ATP9A	6+7+8+9	0	-6.13110181	6.13110181	1.89E-06	7.28E-05	-98.5732284	0.01426772
Q9UI42	CPA4	6+7+8+9	0	-2.96637122	2.96637122	2.18E-06	7.67E-05	-87.2052064	0.12794794
P05089	ARG1	6+7+8+9	0	-1.52973689	1.52973689	2.20E-06	7.67E-05	-65.3659476	0.34634052
Q99536	VAT1	6+7+8+9	0	-0.95081915	0.95081915	2.88E-06	9.46E-05	-48.266136	0.51733864
P50135	HNMT	6+7+8+9	0	1.10355	1.10355	3.13E-06	9.71E-05	114.882799	2.14882799
O95394	PGM3	6+7+8+9	0	-1.27632955	1.27632955	3.83E-06	0.00011279	-58.7155484	0.41284452
Q86V85	GPR180	6+7+8+9	0	-0.99847604	0.99847604	4.53E-06	0.00012822	-49.9471556	0.50052844
P54707	ATP12A	6+7+8+9	0	-2.43095118	2.43095118	4.99E-06	0.00012899	-81.4556858	0.18544314
P16150	SPN	6+7+8+9	0	-1.79203531	1.79203531	5.15E-06	0.00012899	-71.1235622	0.28876438
Q9NQ36	SCUBE2	6+7+8+9	0	-0.9746988	0.9746988	5.53E-06	0.00012899	-49.1153931	0.50884607
Q9BRK3	MXRA8	6+7+8+9	0	-0.90359458	0.90359458	5.10E-06	0.00012899	-46.5446808	0.53455319
P15559	NQO1	6+7+8+9	0	0.83742171	0.83742171	5.65E-06	0.00012899	78.6853943	1.78685394
Q8IWY4	SCUBE1	6+7+8+9	0	-1.10874324	1.10874324	6.06E-06	0.00013034	-53.630221	0.46369779
Q8TF66	LRRC15	6+7+8+9	0	-0.98337584	0.98337584	7.13E-06	0.00014753	-49.4205184	0.50579482
P23381	WARS	6+7+8+9	0	-1.31628915	1.31628915	8.17E-06	0.00016612	-59.8433494	0.40156651
P02679	FGG	6+7+8+9	0	-1.65305877	1.65305877	1.08E-05	0.00021091	-68.2034702	0.3179653

P81605	DCD	6+7+8+9	0	-1.91548339	1.91548339	1.11E-05	0.00021309	-73.4916898	0.2650831
Q9BX67	JAM3	6+7+8+9	0	-1.48222358	1.48222358	1.22E-05	0.00022321	-64.2063291	0.35793671
P54727	RAD23B	6+7+8+9	0	-1.4500542	1.4500542	1.21E-05	0.00022321	-63.3992326	0.36600767
P25705	ATP5F1A	6+7+8+9	0	-2.15475799	2.15475799	1.29E-05	0.00022594	-77.5429238	0.22457076
Q96P63	SERPINB12	6+7+8+9	0	-1.65641614	1.65641614	1.28E-05	0.00022594	-68.2773794	0.31722621
P61769	B2M	6+7+8+9	0	-1.39549865	1.39549865	1.36E-05	0.00023317	-61.9886715	0.38011329
Q8N436	CPXM2	6+7+8+9	0	-1.15108069	1.15108069	1.50E-05	0.00025206	-54.9712195	0.45028781
P08118	MSMB	6+7+8+9	0	-0.94577264	0.94577264	1.66E-05	0.00027076	-48.0848554	0.51915145
P13639	EEF2	6+7+8+9	0	-0.82996137	0.82996137	1.67E-05	0.00027076	-43.7455693	0.56254431
P25786	PSMA1	6+7+8+9	0	-1.09179527	1.09179527	1.83E-05	0.00028434	-53.0822826	0.46917718
Q86TD4	SRL	6+7+8+9	0	-1.22234821	1.22234821	1.92E-05	0.00028983	-57.1415439	0.42858456
Q86YZ3	HRNR	6+7+8+9	0	-1.91192005	1.91192005	1.96E-05	0.00029087	-73.4261356	0.26573864
Q15848	ADIPOQ	6+7+8+9	0	-1.14962261	1.14962261	1.98E-05	0.00029087	-54.9256875	0.45074313
Q02985	CFHR3	6+7+8+9	0	-1.36660786	1.36660786	2.20E-05	0.00030408	-61.2198001	0.387802
Q09327	MGAT3	6+7+8+9	0	-1.31248286	1.31248286	2.23E-05	0.00030408	-59.7372635	0.40262737
P02760	AMBP	6+7+8+9	0	-0.99609663	0.99609663	2.18E-05	0.00030408	-49.8645365	0.50135464
O43405	COCH	6+7+8+9	0	-0.85928099	0.85928099	2.10E-05	0.00030408	-44.8772789	0.55122721
Q6P531	GGT6	6+7+8+9	0	-0.85047209	0.85047209	2.14E-05	0.00030408	-44.5396774	0.55460323
Q92817	EVPL	6+7+8+9	0	-1.64710596	1.64710596	2.43E-05	0.00032684	-68.0720011	0.31927999
P11684	SCGB1A1	6+7+8+9	0	-1.41166907	1.41166907	2.51E-05	0.00032976	-62.412342	0.37587658
P35908	KRT2	6+7+8+9	0	-2.17643227	2.17643227	2.94E-05	0.00036484	-77.8777853	0.22122215
P04264	KRT1	6+7+8+9	0	-1.85965396	1.85965396	3.19E-05	0.00038391	-72.4457639	0.27554236
Q9GZP4	PITHD1	6+7+8+9	0	-0.89753866	0.89753866	3.26E-05	0.00038779	-46.3198228	0.53680177
Q9Y4L1	HYOU1	6+7+8+9	0	-1.48442141	1.48442141	3.37E-05	0.00039259	-64.2608162	0.35739184
Q6UXG3	CD300LG	6+7+8+9	0	-0.86340027	0.86340027	3.36E-05	0.00039259	-45.0344447	0.54965555
P30040	ERP29	6+7+8+9	0	-2.08100954	2.08100954	3.69E-05	0.00040875	-76.3651034	0.23634897
P35858	IGFALS	6+7+8+9	0	-1.10019364	1.10019364	3.61E-05	0.00040875	-53.3546115	0.46645389
P07998	RNASE1	6+7+8+9	0	-1.07019143	1.07019143	3.69E-05	0.00040875	-52.37442	0.4762558
O14818	PSMA7	6+7+8+9	0	-1.04721669	1.04721669	3.69E-05	0.00040875	-51.6099171	0.48390083
Q13867	BLMH	6+7+8+9	0	-1.32484256	1.32484256	3.80E-05	0.00041673	-60.0807245	0.39919276
Q9UGM5	FETUB	6+7+8+9	0	-1.12110742	1.12110742	3.91E-05	0.00042395	-54.0259209	0.45974079
Q9BYE2	TMPRSS13	6+7+8+9	0	-2.34152747	2.34152747	3.98E-05	0.00042798	-80.2698677	0.19730132
P43627	KIR2DL2	6+7+8+9	0	-5.15223311	5.15223311	4.43E-05	0.00045971	-97.1879484	0.02812052
P13646	KRT13	6+7+8+9	0	-2.23512572	2.23512572	4.43E-05	0.00045971	-78.7597261	0.21240274
P20333	TNFRSF1B	6+7+8+9	0	-0.85741276	0.85741276	4.43E-05	0.00045971	-44.8058512	0.55194149
P14923	JUP	6+7+8+9	0	-1.84842914	1.84842914	4.73E-05	0.00047593	-72.2305432	0.27769457
Q8NBS9	TXNDC5	6+7+8+9	0	-0.97889418	0.97889418	4.86E-05	0.00048475	-49.2631513	0.50736849
P29401	TKT	6+7+8+9	0	-0.92917959	0.92917959	5.08E-05	0.00050268	-47.4843105	0.5251569
Q08554	DSC1	6+7+8+9	0	-1.82654095	1.82654095	5.39E-05	0.00051965	-71.8060202	0.2819398

P13645	KRT10	6+7+8+9	0	-2.31249174	2.31249174	5.45E-05	0.00052052	-79.8687555	0.20131245
Q9Y5Y7	LYVE1	6+7+8+9	0	-0.98126351	0.98126351	5.98E-05	0.00056648	-49.3464078	0.50653592
Q5D862	FLG2	6+7+8+9	0	-2.21421826	2.21421826	6.19E-05	0.00056687	-78.4496718	0.21550328
P04003	C4BPA	6+7+8+9	0	-0.92630135	0.92630135	6.12E-05	0.00056687	-47.3794346	0.52620565
Q6UWH4	GASK1B	6+7+8+9	0	-1.01865594	1.01865594	6.94E-05	0.0006163	-50.6424032	0.49357597
P80723	BASP1	6+7+8+9	0	-0.86833211	0.86833211	7.28E-05	0.00064047	-45.222023	0.54777977
P02533	KRT14	6+7+8+9	0	-1.97213393	1.97213393	7.64E-05	0.00065166	-74.5124241	0.25487576
P33176	KIF5B	6+7+8+9	0	-1.32002342	1.32002342	8.43E-05	0.00069335	-59.9471564	0.40052844
P49913	CAMP	6+7+8+9	0	-0.99729886	0.99729886	8.61E-05	0.00070242	-49.9062979	0.50093702
P62495	ETF1	6+7+8+9	0	-1.31455933	1.31455933	9.13E-05	0.00072402	-59.7951718	0.40204828
Q02413	DSG1	6+7+8+9	0	-0.93102838	0.93102838	9.85E-05	0.00077018	-47.5515655	0.52448435
O60701	UGDH	6+7+8+9	0	-1.22838407	1.22838407	0.0001113	0.00085227	-57.3204777	0.42679522
O00264	PGRMC1	6+7+8+9	0	-1.19816529	1.19816529	0.00011276	0.0008576	-56.4170817	0.43582918
P35030	PRSS3	6+7+8+9	0	-1.58482051	1.58482051	0.00011569	0.00087391	-66.6633858	0.33336614
O95969	SCGB1D2	6+7+8+9	0	-1.27038407	1.27038407	0.00012896	0.00092421	-58.5450601	0.4145494
Q5T749	KPRP	6+7+8+9	0	-1.73114971	1.73114971	0.00014523	0.00102118	-69.8788181	0.30121182
P10645	CHGA	6+7+8+9	0	-0.81972868	0.81972868	0.00014741	0.00103006	-43.3451521	0.56654848
P34913	EPHX2	6+7+8+9	0	-0.98973186	0.98973186	0.00015017	0.00103154	-49.6428639	0.50357136
P07477	PRSS1	6+7+8+9	0	-2.02301678	2.02301678	0.00015657	0.00105932	-75.3956856	0.24604314
P41439	FOLR3	6+7+8+9	0	-1.67410045	1.67410045	0.00015671	0.00105932	-68.6638564	0.31336144
P25311	AZGP1	6+7+8+9	0	-0.80919156	0.80919156	0.00015729	0.00105932	-42.929843	0.57070157
P35527	KRT9	6+7+8+9	0	-1.97020745	1.97020745	0.0001638	0.00107096	-74.478367	0.25521633
O75342	ALOX12B	6+7+8+9	0	-1.12267308	1.12267308	0.0001691	0.00109913	-54.0757865	0.45924214
O43511	SLC26A4	6+7+8+9	0	-1.5155017	1.5155017	0.00017722	0.00113219	-65.0225189	0.34977481
P30408	TM4SF1	6+7+8+9	0	-1.11460924	1.11460924	0.00018137	0.0011521	-53.8183776	0.46181622
O75023	LILRB5	6+7+8+9	0	-0.86590983	0.86590983	0.00018951	0.00119031	-45.1299737	0.54870026
A0A0A0MRZ8; P04433	IGKV3D-11;IGKV3-11	6+7+8+9	0	-0.8408342	0.8408342	0.00019569	0.00121547	-44.167936	0.55832064
P16671	CD36	6+7+8+9	0	-3.41845728	3.41845728	0.00019834	0.00121707	-90.6471963	0.09352804
P55017	SLC12A3	6+7+8+9	0	-3.04336872	3.04336872	0.00019922	0.00121707	-87.8701696	0.1212983
P69905	HBA1	6+7+8+9	0	-1.26523929	1.26523929	0.00019738	0.00121707	-58.3969643	0.41603036
P22735	TGM1	6+7+8+9	0	-1.55645984	1.55645984	0.00021327	0.00128886	-66.0015671	0.33998433
P01023	A2M	6+7+8+9	0	-1.02071889	1.02071889	0.00022	0.00131528	-50.7129305	0.4928707
P43626	KIR2DL1	6+7+8+9	0	-1.07365414	1.07365414	0.00022192	0.00131972	-52.4885923	0.47511408
Q8NBJ7	SUMF2	6+7+8+9	0	-0.98286778	0.98286778	0.00022681	0.00133924	-49.402703	0.50597297
Q8N335	GPD1L	6+7+8+9	0	-0.84215912	0.84215912	0.0002276	0.00133924	-44.2191863	0.55780814
P21453	S1PR1	6+7+8+9	0	-1.00071781	1.00071781	0.00023062	0.00134993	-50.0248713	0.49975129
Q9HB21	PLEKHA1	6+7+8+9	0	1.09912799	1.09912799	0.0002326	0.00135369	114.225168	2.14225168
P49720	PSMB3	6+7+8+9	0	-2.41355324	2.41355324	0.00023985	0.00136116	-81.2306999	0.187693

O95865	DDAH2	6+7+8+9	0	-0.91765417	0.91765417	0.00026104	0.00144478	-47.0630923	0.52936908
Q92823	NRCAM	6+7+8+9	0	-2.71252257	2.71252257	0.00029226	0.00159386	-84.7436957	0.15256304
P02810	PRH1	6+7+8+9	0	-2.93119914	2.93119914	0.00029954	0.00161778	-86.8894432	0.13110557
P31939	ATIC	6+7+8+9	0	-1.55583623	1.55583623	0.00030375	0.00162484	-65.9868679	0.34013132
Q96NZ9	PRAP1	6+7+8+9	0	-1.74854284	1.74854284	0.00031075	0.00163876	-70.2397786	0.29760221
Q15485	FCN2	6+7+8+9	0	-0.95935031	0.95935031	0.00031028	0.00163876	-48.5711539	0.51428846
Q9BQI0	AIF1L	6+7+8+9	0	0.98298209	0.98298209	0.00031638	0.00165209	97.6546766	1.97654677
Q7Z794	KRT77	6+7+8+9	0	-1.67897499	1.67897499	0.00034119	0.00171051	-68.7695554	0.31230445
P02747	C1QC	6+7+8+9	0	-1.47101492	1.47101492	0.00033934	0.00171051	-63.9271558	0.36072844
P06899;P23527;P33778;Q16778;Q8N257	HIST1H2BJ;HIST1H2BO;HIST1H2BB;HIST2H2BE;HIST3H2BB	6+7+8+9	0	-0.91646459	0.91646459	0.00035537	0.00177366	-47.0194247	0.52980575
P13647	KRT5	6+7+8+9	0	-1.77409114	1.77409114	0.00036104	0.00179399	-70.7621556	0.29237844
P60983	GMFB	6+7+8+9	0	-0.95674926	0.95674926	0.00036562	0.00180869	-48.4783488	0.51521651
Q13261	IL15RA	6+7+8+9	0	-1.00819655	1.00819655	0.00041316	0.00199099	-50.2832653	0.49716735
Q99733	NAP1L4	6+7+8+9	0	1.03375875	1.03375875	0.0004271	0.00204057	104.735141	2.04735141
Q5T750	XP32	6+7+8+9	0	-1.47473472	1.47473472	0.00044592	0.00211245	-64.0200453	0.35979955
P60900	PSMA6	6+7+8+9	0	-0.98181219	0.98181219	0.00044918	0.00211889	-49.3656684	0.50634332
Q7Z2H8	SLC36A1	6+7+8+9	0	-0.86113919	0.86113919	0.00045519	0.00213825	-44.9482317	0.55051768
P51858	HDGF	6+7+8+9	0	-1.01236572	1.01236572	0.00046202	0.00216125	-50.4267317	0.49573268
P10163	PRB4	6+7+8+9	0	-5.8685724	5.8685724	0.00047748	0.00221503	-98.2884733	0.01711527
Q96QK1	VPS35	6+7+8+9	0	-1.09779041	1.09779041	0.00048528	0.00223268	-53.2768452	0.46723155
P48740	MASP1	6+7+8+9	0	-1.41131062	1.41131062	0.00052625	0.00234403	-62.4030018	0.37596998
Q8NCH0	CHST14	6+7+8+9	0	2.58509096	2.58509096	0.00054309	0.00240941	500.053426	6.00053426
Q9ULV0	MYO5B	6+7+8+9	0	-3.85918515	3.85918515	0.000572	0.00248185	-93.1092021	0.06890798
P02538	KRT6A	6+7+8+9	0	-1.93468077	1.93468077	0.00057295	0.00248185	-73.8420891	0.26157911
P12830	CDH1	6+7+8+9	0	-1.35472552	1.35472552	0.0005794	0.00248185	-60.8990793	0.39100921
O00461	GOLIM4	6+7+8+9	0	-0.81341637	0.81341637	0.00057831	0.00248185	-43.0967233	0.56903277
Q06323	PSME1	6+7+8+9	0	-4.53991194	4.53991194	0.00060421	0.00256468	-95.7011694	0.04298831
Q9UKZ9	PCOLCE2	6+7+8+9	0	-0.85469005	0.85469005	0.00064322	0.00270346	-44.7015881	0.55298412
P08F94	PKHD1	6+7+8+9	0	-1.19734624	1.19734624	0.0006527	0.00270364	-56.3923315	0.43607669
P19801	AOC1	6+7+8+9	0	-1.16509464	1.16509464	0.00064868	0.00270364	-55.4064995	0.44593501
P62906	RPL10A	6+7+8+9	0	-4.7822941	4.7822941	0.00065685	0.00270979	-96.3659908	0.03634009
Q6UWR7	ENPP6	6+7+8+9	0	-1.46997932	1.46997932	0.00066085	0.00271453	-63.9012525	0.36098748
Q9Y5X3	SNX5	6+7+8+9	0	-1.97531116	1.97531116	0.00067436	0.00274156	-74.5684934	0.25431507
P22532;P35325	SPRR2D;SPRR2B	6+7+8+9	0	-0.89269124	0.89269124	0.00068358	0.00275899	-46.1391554	0.53860845
P49411	TUFM	6+7+8+9	0	2.95181718	2.95181718	0.00068747	0.00276098	673.723012	7.73723012
Q14210	LY6D	6+7+8+9	0	-1.23597938	1.23597938	0.00068901	0.00276098	-57.5445808	0.42455419
P08779	KRT16	6+7+8+9	0	-1.41201644	1.41201644	0.00070929	0.00283209	-62.4213914	0.37578609

Q15828	CST6	6+7+8+9	0	-1.04962699	1.04962699	0.00071227	0.00283388	-51.6906947	0.48309305
P05109	S100A8	6+7+8+9	0	-1.10287027	1.10287027	0.00071995	0.00285428	-53.4410726	0.46558927
P47972	NPTX2	6+7+8+9	0	-0.98097598	0.98097598	0.00074232	0.00291199	-49.3363115	0.50663689
Q9UHC6	CNTNAP2	6+7+8+9	0	-1.10381795	1.10381795	0.00075877	0.00295576	-53.4716461	0.46528354
P12319	FCER1A	6+7+8+9	0	-0.9356869	0.9356869	0.00078269	0.00303836	-47.7206502	0.5227935
P09564	CD7	6+7+8+9	0	-0.92535995	0.92535995	0.00081861	0.00312378	-47.3450871	0.52654913
P31151	S100A7	6+7+8+9	0	-2.06173409	2.06173409	0.00087037	0.00327635	-76.047205	0.23952795
P35270	SPR	6+7+8+9	0	-2.80069288	2.80069288	0.00088488	0.00328671	-85.6481649	0.14351835
P15924	DSP	6+7+8+9	0	-1.79799221	1.79799221	0.00088254	0.00328671	-71.2425474	0.28757453
P49447	CYB561	6+7+8+9	0	-0.84645861	0.84645861	0.00090294	0.00330981	-44.3851758	0.55614824
P07237	P4HB	6+7+8+9	0	-6.76524904	6.76524904	0.00093639	0.00337889	-99.0807003	0.009193
O15511	ARPC5	6+7+8+9	0	-0.86435756	0.86435756	0.00093689	0.00337889	-45.0709046	0.54929095
Q7Z3Y8	KRT27	6+7+8+9	0	-2.65688635	2.65688635	0.00095537	0.0034234	-84.1438585	0.15856142
P00915	CA1	6+7+8+9	0	-1.36600569	1.36600569	0.00096952	0.00345197	-61.2036103	0.3879639
Q9NZT1	CALML5	6+7+8+9	0	-1.31549423	1.31549423	0.00099266	0.00347896	-59.821217	0.40178783
P08174	CD55	6+7+8+9	0	-0.81175972	0.81175972	0.00098167	0.00347896	-43.0313437	0.56968656
Q9H4G4	GLIPR2	6+7+8+9	0	-0.93920324	0.93920324	0.00101774	0.00353366	-47.8479177	0.52152082
A0A075B6P5;<br>P01615	IGKV2-28;IGKV2D-28	6+7+8+9	0	-1.01998307	1.01998307	0.00102504	0.00354797	-50.6877861	0.49312214
Q9HCY8	S100A14	6+7+8+9	0	-1.05146546	1.05146546	0.00106668	0.00362476	-51.7522175	0.48247783
Q14247	CTTN	6+7+8+9	0	-0.86078258	0.86078258	0.0010724	0.00363315	-44.9346223	0.55065378
P48304	REG1B	6+7+8+9	0	-1.5158428	1.5158428	0.00109906	0.00367887	-65.0307877	0.34969212
Q5JTV8	TOR1AIP1	6+7+8+9	0	-1.45533806	1.45533806	0.00110856	0.0036886	-63.5330375	0.36466963
P10644	PRKAR1A	6+7+8+9	0	-2.01521865	2.01521865	0.00111397	0.00369559	-75.2623331	0.24737667
P02814	SMR3B	6+7+8+9	0	-2.25954473	2.25954473	0.00113025	0.00372751	-79.1162128	0.20883787
P55283	CDH4	6+7+8+9	0	1.29244267	1.29244267	0.00120313	0.00387636	144.942424	2.44942424
P50443	SLC26A2	6+7+8+9	0	-1.35952922	1.35952922	0.00122267	0.0039254	-61.0290561	0.38970944
Q9Y561	LRP12	6+7+8+9	0	-1.93383362	1.93383362	0.00127864	0.00405466	-73.8267247	0.26173275
P42892	ECE1	6+7+8+9	0	-1.85052174	1.85052174	0.00129305	0.00407528	-72.2707931	0.27729207
P28066	PSMA5	6+7+8+9	0	-0.81173841	0.81173841	0.00129403	0.00407528	-43.0305024	0.56969498
P43686	PSMC4	6+7+8+9	0	-3.2900349	3.2900349	0.0013456	0.00421396	-89.7764716	0.10223528
P35240	NF2	6+7+8+9	0	1.33961725	1.33961725	0.00140067	0.00436198	153.084165	2.53084165
P19013	KRT4	6+7+8+9	0	-2.25687256	2.25687256	0.00141631	0.00437414	-79.0774958	0.20922504
P29966	MARCKS	6+7+8+9	0	-0.88479746	0.88479746	0.00142921	0.00438571	-45.8436454	0.54156355
P53004	BLVRA	6+7+8+9	0	-3.21105635	3.21105635	0.00144016	0.00438718	-89.2011945	0.10798806
P00492	HPRT1	6+7+8+9	0	-0.81904503	0.81904503	0.00145182	0.00439874	-43.3182986	0.56681701
P02042	HBD	6+7+8+9	0	-2.63780045	2.63780045	0.00147212	0.00444819	-83.9326986	0.16067301
Q9HC84	MUC5B	6+7+8+9	0	-2.3909768	2.3909768	0.00148474	0.00447424	-80.9346728	0.19065327
P25789	PSMA4	6+7+8+9	0	-1.25192473	1.25192473	0.00162199	0.00476423	-58.0112348	0.41988765

P28906	CD34	6+7+8+9	0	-1.00150229	1.00150229	0.00163333	0.00476423	-50.0520382	0.49947962
P80511	S100A12	6+7+8+9	0	-1.19678009	1.19678009	0.00166839	0.00480738	-56.3752154	0.43624785
P02730	SLC4A1	6+7+8+9	0	-0.83736486	0.83736486	0.00166084	0.00480738	-44.0335114	0.55966489
Q8IUE6	HIST2H2AB	6+7+8+9	0	-2.34981322	2.34981322	0.00169864	0.00488194	-80.382858	0.19617142
Q9BWD1	ACAT2	6+7+8+9	0	-1.75792858	1.75792858	0.00172714	0.00493848	-70.4327613	0.29567239
P20472	PVALB	6+7+8+9	0	1.03449617	1.03449617	0.0017449	0.00496387	104.839816	2.04839816
A6NCW0;A8MUK1;C9J2P7;C9JJH3;C9JLJ4;C9JPN9;C9JVI0;D6R901;D6R9N7;D6RA61;D6RBQ6;D6RCP7;D6RJB6;Q0WX57;Q7RTZ2	USP17L3;USP17L5;USP17L15;USP17L10;USP17L13;USP17L12;USP17L11;USP17L21;USP17L18;USP17L22;USP17L17;USP17L19;USP17L20;USP17L24;USP17L1	6+7+8+9	0	-1.63346493	1.63346493	0.0018312	0.0050801	-67.7686825	0.32231318
O00244	ATOX1	6+7+8+9	0	-1.24094454	1.24094454	0.00182277	0.0050801	-57.6904436	0.42309556
P11216	PYGB	6+7+8+9	0	-0.85389015	0.85389015	0.00182069	0.0050801	-44.6709197	0.5532908
P07359	GP1BA	6+7+8+9	0	-1.02010507	1.02010507	0.00184035	0.00509285	-50.691956	0.49308044
P05198	EIF2S1	6+7+8+9	0	-6.01356735	6.01356735	0.00213343	0.00555986	-98.4521251	0.01547875
Q99747	NAPG	6+7+8+9	0	-0.98088768	0.98088768	0.0021909	0.00569635	-49.3332104	0.5066679
Q15631	TSN	6+7+8+9	0	-1.30037203	1.30037203	0.00222835	0.00578026	-59.3978517	0.40602148
Q9NT68;Q9P273	TENM2;TENM3	6+7+8+9	0	-1.62387728	1.62387728	0.0023173	0.00598323	-67.5537709	0.32446229
P30453	HLA-A	6+7+8+9	0	-0.88464944	0.88464944	0.00238784	0.00612923	-45.8380886	0.54161911
P50995	ANXA11	6+7+8+9	0	-0.91795398	0.91795398	0.00252997	0.00642843	-47.0740922	0.52925908
P25788	PSMA3	6+7+8+9	0	-1.3732171	1.3732171	0.00275091	0.00685607	-61.3970527	0.38602947
Q687X5	STEAP4	6+7+8+9	0	-0.97841271	0.97841271	0.00275109	0.00685607	-49.2462161	0.50753784
Q6ISS4	LAIR2	6+7+8+9	0	-0.81565244	0.81565244	0.00275727	0.00685607	-43.184851	0.56815149
O95436	SLC34A2	6+7+8+9	0	6.98824302	6.98824302	0.00286059	0.00705323	12596.1126	126.961126
P29508	SERPINB3	6+7+8+9	0	-1.9075516	1.9075516	0.00285159	0.00705323	-73.3455485	0.26654452
P61313	RPL15	6+7+8+9	0	-0.9840396	0.9840396	0.0029158	0.00713319	-49.4437837	0.50556216
Q9UL46	PSME2	6+7+8+9	0	-1.09241324	1.09241324	0.0029925	0.00725731	-53.1023752	0.46897625
O60883	GPR37L1	6+7+8+9	0	-2.44310938	2.44310938	0.00305544	0.00737377	-81.6113098	0.1838869
Q9UBQ7	GRHPR	6+7+8+9	0	-0.87947036	0.87947036	0.00311586	0.00747539	-45.6433052	0.54356695
P21730	C5AR1	6+7+8+9	0	-1.08436852	1.08436852	0.00321225	0.00765735	-52.8401354	0.47159865
P47929	LGALS7	6+7+8+9	0	-0.88738616	0.88738616	0.00328569	0.00779916	-45.9407335	0.54059267
Q04695	KRT17	6+7+8+9	0	-1.01664177	1.01664177	0.00339194	0.00800039	-50.5734461	0.49426554
Q6ZP82	CCDC141	6+7+8+9	0	-2.58669029	2.58669029	0.00347774	0.00818549	-83.3532816	0.16646718
Q96MG2	JSRP1	6+7+8+9	0	-0.82198656	0.82198656	0.00354925	0.00833626	-43.4337498	0.5656625
P0CG47;P0CG48;P62979;P62987	UBB;UBC;RPS27A;UBA52	6+7+8+9	0	-1.13255735	1.13255735	0.00361533	0.00843828	-54.3893494	0.45610651

O00231	PSMD11	6+7+8+9	0	2.1270844	2.1270844	0.00365111	0.00847755	336.833773	4.36833773
Q15262	PTPRK	6+7+8+9	0	-1.96060581	1.96060581	0.00369996	0.0085584	-74.307945	0.25692055
P40227	CCT6A	6+7+8+9	0	-1.72832732	1.72832732	0.00375559	0.00862414	-69.8198333	0.30180167
P05534	HLA-A	6+7+8+9	0	-1.05442079	1.05442079	0.00377648	0.00862414	-51.8509508	0.48149049
Q8N9U0	TC2N	6+7+8+9	0	-0.82431825	0.82431825	0.00377981	0.00862414	-43.5250985	0.56474902
P09488	GSTM1	6+7+8+9	0	-1.01235749	1.01235749	0.00389343	0.00881144	-50.4264488	0.49573551
O60449	LY75	6+7+8+9	0	-0.9575473	0.9575473	0.0038865	0.00881144	-48.5068403	0.5149316
Q14152	EIF3A	6+7+8+9	0	3.29987016	3.29987016	0.00393467	0.00886887	884.826893	9.84826893
P23490	LOR	6+7+8+9	0	-1.61750703	1.61750703	0.00400974	0.00901991	-67.4101873	0.32589813
Q12905	ILF2	6+7+8+9	0	1.12790172	1.12790172	0.00413258	0.00924045	118.54066	2.1854066
P05186	ALPL	6+7+8+9	0	-1.4391791	1.4391791	0.00419822	0.00934983	-63.1222919	0.36877708
P48552	NRIP1	6+7+8+9	0	-1.18135763	1.18135763	0.00440897	0.00976085	-55.9063635	0.44093637
P29034	S100A2	6+7+8+9	0	-1.61207818	1.61207818	0.00458473	0.01009001	-67.2873209	0.32712679
Q9BYG5	PARD6B	6+7+8+9	0	2.83884051	2.83884051	0.00481869	0.01038014	615.444824	7.15444824
Q86UX2	ITIH5	6+7+8+9	0	-1.13852729	1.13852729	0.00483668	0.01039887	-54.5776985	0.45422302
P41227	NAA10	6+7+8+9	0	-1.59735144	1.59735144	0.00494842	0.01059718	-66.9516864	0.33048314
Q9UKX2	MYH2	6+7+8+9	0	-5.46850721	5.46850721	0.00499698	0.01064118	-97.741525	0.02258475
P15309	ACPP	6+7+8+9	0	-1.15766662	1.15766662	0.00507491	0.01076613	-55.1763083	0.44823692
P20774	OGN	6+7+8+9	0	-0.95485303	0.95485303	0.00507158	0.01076613	-48.4105858	0.51589414
P17612	PRKACA	6+7+8+9	0	-1.34860171	1.34860171	0.00530959	0.01113718	-60.7327549	0.39267245
P07203	GPX1	6+7+8+9	0	-4.00509979	4.00509979	0.00537848	0.01123951	-93.7720542	0.06227946
P26012	ITGB8	6+7+8+9	0	-3.32399974	3.32399974	0.00539596	0.01125192	-90.0143492	0.09985651
A0A0A0MT36	IGKV6D-21	6+7+8+9	0	-2.33705726	2.33705726	0.00550771	0.0114454	-80.2086389	0.19791361
Q9BYF1	ACE2	6+7+8+9	0	-4.86100922	4.86100922	0.00551984	0.01144932	-96.5589545	0.03441046
P52209	PGD	6+7+8+9	0	-0.97437985	0.97437985	0.00567213	0.01165705	-49.1041423	0.50895858
Q9NR45	NANS	6+7+8+9	0	-0.9938014	0.9938014	0.0057164	0.01170786	-49.7847108	0.50215289
Q13316	DMP1	6+7+8+9	0	-0.9650228	0.9650228	0.00571779	0.01170786	-48.7729684	0.51227032
P12532;P17540	CKMT1A;CKMT2	6+7+8+9	0	-1.15023801	1.15023801	0.0058776	0.0119206	-54.9449106	0.45055089
P34741	SDC2	6+7+8+9	0	-1.42293788	1.42293788	0.00647108	0.01276181	-62.7047935	0.37295207
P01715	IGLV3-1	6+7+8+9	0	-1.07210017	1.07210017	0.00673729	0.01314536	-52.4373887	0.47562611
P04259	KRT6B	6+7+8+9	0	-1.68256622	1.68256622	0.0068105	0.01326506	-68.8471992	0.31152801
Q13530	SERINC3	6+7+8+9	0	-1.79442185	1.79442185	0.0068739	0.01334204	-71.1712908	0.28828709
Q8N4F0	BPIFB2	6+7+8+9	0	-2.20332406	2.20332406	0.00712386	0.01370821	-78.2863232	0.21713677
Q9BSE5	AGMAT	6+7+8+9	0	1.95194218	1.95194218	0.00728615	0.01399642	286.895026	3.86895026
Q9Y6U3	SCIN	6+7+8+9	0	-1.04160738	1.04160738	0.007432	0.01422769	-51.4214068	0.48578593
Q86UN2	RTN4RL1	6+7+8+9	0	-1.56129316	1.56129316	0.00758261	0.01444184	-66.115278	0.33884722
Q6UXF1	TMEM108	6+7+8+9	0	-0.93372486	0.93372486	0.00790089	0.01494618	-47.6495029	0.52350497
Q53TN4	CYBRD1	6+7+8+9	0	-0.9481291	0.9481291	0.00817877	0.01521287	-48.1695831	0.51830417
P06310	IGKV2-30	6+7+8+9	0	-1.03902765	1.03902765	0.00837526	0.01538905	-51.3344641	0.48665536

P07288	KLK3	6+7+8+9	0	-0.81778252	0.81778252	0.00838152	0.01538905	-43.2686743	0.56731326
P23458	JAK1	6+7+8+9	0	-1.71569155	1.71569155	0.0084571	0.01550007	-69.5543409	0.30445659
Q9BRF8	CPPED1	6+7+8+9	0	-0.95745709	0.95745709	0.0087151	0.01586887	-48.5036206	0.51496379
P02746	C1QB	6+7+8+9	0	-0.87515894	0.87515894	0.00882305	0.0160393	-45.4806201	0.5451938
P04040	CAT	6+7+8+9	0	-1.0907538	1.0907538	0.00911086	0.01648211	-53.0484008	0.46951599
Q9UNW1	MINPP1	6+7+8+9	0	-0.9041945	0.9041945	0.00930143	0.01677258	-46.5669047	0.53433095
Q96TA1	FAM129B	6+7+8+9	0	-2.00233132	2.00233132	0.00971436	0.01732161	-75.0403661	0.24959634
Q9Y2J2	EPB41L3	6+7+8+9	0	-1.01233919	1.01233919	0.01008771	0.01782482	-50.42582	0.4957418
P04114	APOB	6+7+8+9	0	-2.49068253	2.49068253	0.01014054	0.01782566	-82.207792	0.17792208
O95971	CD160	6+7+8+9	0	-1.10596229	1.10596229	0.01013542	0.01782566	-53.540752	0.46459248
P00505	GOT2	6+7+8+9	0	0.83796762	0.83796762	0.01012516	0.01782566	78.7530209	1.78753021
Q8IVY1	C1orf210	6+7+8+9	0	1.78351294	1.78351294	0.01035733	0.01809296	244.26343	3.4426343
Q96H20	SNF8	6+7+8+9	0	-3.90891528	3.90891528	0.01057486	0.01830138	-93.3426828	0.06657317
Q14112	NID2	6+7+8+9	0	-0.85377705	0.85377705	0.01111065	0.01884933	-44.6665818	0.55333418
P78371	CCT2	6+7+8+9	0	-2.17057669	2.17057669	0.01121512	0.01894033	-77.7878136	0.22212186
P29373	CRABP2	6+7+8+9	0	-1.48610536	1.48610536	0.01143206	0.01921961	-64.3025075	0.35697493
P38646	HSPA9	6+7+8+9	0	-2.37861916	2.37861916	0.01159557	0.01929645	-80.7706641	0.19229336
P08476	INHBA	6+7+8+9	0	1.49880754	1.49880754	0.01164162	0.01933928	182.609024	2.82609024
P00451	F8	6+7+8+9	0	-3.92514001	3.92514001	0.01189044	0.01963591	-93.4171324	0.06582868
P84098	RPL19	6+7+8+9	0	-1.64022564	1.64022564	0.01249074	0.02033502	-67.9193703	0.3208063
P22626	HNRNPA2B1	6+7+8+9	0	-2.25098276	2.25098276	0.01253523	0.02036975	-78.9919052	0.21008095
A0A075B6H9	IGLV4-69	6+7+8+9	0	-1.02040214	1.02040214	0.01259272	0.02043347	-50.7021082	0.49297892
P43490	NAMPT	6+7+8+9	0	-1.09428553	1.09428553	0.01328552	0.0213102	-53.1631983	0.46836802
Q99102	MUC4	6+7+8+9	0	-0.8174897	0.8174897	0.01351574	0.02164118	-43.2571587	0.56742841
Q13443	ADAM9	6+7+8+9	0	-1.56505636	1.56505636	0.01378129	0.02185044	-66.2035496	0.3379645
O00571	DDX3X	6+7+8+9	0	-0.99973707	0.99973707	0.01431798	0.02241947	-49.9908867	0.50009113
P40305	IFI27	6+7+8+9	0	2.26505438	2.26505438	0.01453076	0.02268909	380.672537	4.80672537
P30740	SERPINB1	6+7+8+9	0	-1.36054232	1.36054232	0.01552575	0.02379237	-61.0564129	0.38943587
Q96QA5	GSDMA	6+7+8+9	0	-2.27659148	2.27659148	0.01597009	0.02433024	-79.3615215	0.20638479
Q10567	AP1B1	6+7+8+9	0	2.90452146	2.90452146	0.0162645	0.02463918	648.76939	7.4876939
P25445	FAS	6+7+8+9	0	-2.02941972	2.02941972	0.0162481	0.02463918	-75.504642	0.24495358
P24298	GPT	6+7+8+9	0	-1.45634943	1.45634943	0.01654618	0.02489721	-63.5585929	0.36441407
Q9ULK6	RNF150	6+7+8+9	0	-3.10559399	3.10559399	0.01659977	0.02494428	-88.3822225	0.11617778
P15880	RPS2	6+7+8+9	0	-0.89537416	0.89537416	0.01772347	0.02617549	-46.2392251	0.53760775
Q8WWT9	SLC13A3	6+7+8+9	0	-1.26289221	1.26289221	0.0178878	0.0263139	-58.3292262	0.41670774
P12081	HARS	6+7+8+9	0	-0.82237319	0.82237319	0.01794166	0.02635844	-43.448907	0.56551093
P20933	AGA	6+7+8+9	0	-0.96763149	0.96763149	0.01813189	0.02646404	-48.8655136	0.51134486
P11940	PABPC1	6+7+8+9	0	-1.44738873	1.44738873	0.01877649	0.02712489	-63.3315479	0.36668452
P02776	PF4	6+7+8+9	0	-1.21626497	1.21626497	0.01894785	0.02726344	-56.9604462	0.43039554

Q96C19	EFHD2	6+7+8+9	0	-3.37719485	3.37719485	0.01929203	0.0276519	-90.3758352	0.09624165
Q6H9L7	ISM2	6+7+8+9	0	-1.20944282	1.20944282	0.01993949	0.02832574	-56.7564406	0.43243559
Q13162	PRDX4	6+7+8+9	0	-1.0499318	1.0499318	0.02021928	0.02861412	-51.7009005	0.482991
P30153	PPP2R1A	6+7+8+9	0	-1.34186785	1.34186785	0.02045825	0.02891571	-60.5490444	0.39450956
Q14134	TRIM29	6+7+8+9	0	-1.73387474	1.73387474	0.02112146	0.02940697	-69.9356585	0.30064342
P53384	NUBP1	6+7+8+9	0	1.51359885	1.51359885	0.02106149	0.02940697	185.521394	2.85521394
P02786	TFRC	6+7+8+9	0	-1.40369499	1.40369499	0.02121177	0.02949597	-62.2040119	0.37795988
P18084	ITGB5	6+7+8+9	0	-0.83402957	0.83402957	0.02175555	0.03005345	-43.9039758	0.56096024
P80303	NUCB2	6+7+8+9	0	-1.2327814	1.2327814	0.02198919	0.03023851	-57.4503667	0.42549633
P13500	CCL2	6+7+8+9	0	-1.93533591	1.93533591	0.0222056	0.03042385	-73.8539649	0.26146035
A0A075B6I1	IGLV4-60	6+7+8+9	0	-1.6231016	1.6231016	0.02235179	0.03054984	-67.5363213	0.32463679
Q99952	PTPN18	6+7+8+9	0	1.0978562	1.0978562	0.02247805	0.03068433	114.036405	2.14036405
P04083	ANXA1	6+7+8+9	0	-1.16501309	1.16501309	0.02458554	0.03264445	-55.4039786	0.44596021
P05164	MPO	6+7+8+9	0	-0.848105	0.848105	0.02458322	0.03264445	-44.4486069	0.55551393
Q06210	GFPT1	6+7+8+9	0	-0.93563948	0.93563948	0.02477509	0.03277651	-47.718932	0.52281068
O15400	STX7	6+7+8+9	0	-1.11529385	1.11529385	0.02505508	0.03284278	-53.8402872	0.46159713
Q9H2M3	BHMT2	6+7+8+9	0	-0.88828728	0.88828728	0.02610707	0.03386045	-45.9744889	0.54025511
O43760	SYNGR2	6+7+8+9	0	-0.88264131	0.88264131	0.02687633	0.03457737	-45.7626464	0.54237354
Q86VN1	VPS36	6+7+8+9	0	-1.28291151	1.28291151	0.02736072	0.03503927	-58.90347	0.4109653
Q02790	FKBP4	6+7+8+9	0	-1.46693067	1.46693067	0.02897689	0.03677203	-63.8248893	0.36175111
Q9UHB9	SRP68	6+7+8+9	0	1.35473832	1.35473832	0.02910599	0.03685221	155.750722	2.55750722
A0A075B6I0	IGLV8-61	6+7+8+9	0	-1.13392912	1.13392912	0.02919384	0.03692162	-54.4326971	0.45567303
Q7Z304	MAMDC2	6+7+8+9	0	-3.01449831	3.01449831	0.02952497	0.0371304	-87.6249892	0.12375011
Q8TF65	GIPC2	6+7+8+9	0	-0.99159802	0.99159802	0.02950455	0.0371304	-49.7079599	0.5029204
Q99674	CGREF1	6+7+8+9	0	-0.82539716	0.82539716	0.02949199	0.0371304	-43.5673171	0.56432683
Q08188	TGM3	6+7+8+9	0	-1.27434504	1.27434504	0.02960233	0.03718584	-58.6587201	0.4134128
Q6QNY0	BLOC1S3	6+7+8+9	0	1.95880067	1.95880067	0.02986698	0.0374376	288.738681	3.88738681
P02652	APOA2	6+7+8+9	0	-0.91636572	0.91636572	0.03033982	0.03781485	-47.015794	0.52984206
Q96FN5	KIF12	6+7+8+9	0	-0.92883889	0.92883889	0.03134299	0.03846483	-47.4719072	0.52528093
Q9Y266	NUDC	6+7+8+9	0	-2.77934697	2.77934697	0.03138981	0.03848005	-85.4342386	0.14565762
P35475	IDUA	6+7+8+9	0	-0.93362228	0.93362228	0.03150253	0.03857594	-47.6457805	0.5235422
O00194	RAB27B	6+7+8+9	0	-0.92774108	0.92774108	0.03167297	0.03874221	-47.431921	0.52568079
P03973	SLPI	6+7+8+9	0	-1.5726765	1.5726765	0.03189622	0.03888765	-66.3815876	0.33618412
P06748	NPM1	6+7+8+9	0	-1.58577124	1.58577124	0.03230275	0.03929342	-66.6853473	0.33314653
Q14203	DCTN1	6+7+8+9	0	-0.98541498	0.98541498	0.03237424	0.03929903	-49.4919578	0.50508042
Q96JI7	SPG11	6+7+8+9	0	1.96706688	1.96706688	0.03303791	0.03983212	290.97243	3.9097243
Q9BQB4	SOST	6+7+8+9	0	-0.84974685	0.84974685	0.033122	0.03983212	-44.5117907	0.55488209
P08236	GUSB	6+7+8+9	0	-2.30002262	2.30002262	0.03366664	0.04025982	-79.6940084	0.20305992
P02788	LTF	6+7+8+9	0	-1.04821047	1.04821047	0.03421482	0.0406938	-51.6432386	0.48356761

P55209	NAP1L1	6+7+8+9	0	-1.25292263	1.25292263	0.03442791	0.04073059	-58.0402678	0.41959732
Q13093	PLA2G7	6+7+8+9	0	-0.91096753	0.91096753	0.03438872	0.04073059	-46.8171696	0.5318283
O75844	ZMPSTE24	6+7+8+9	0	-1.00738521	1.00738521	0.03458782	0.04079264	-50.255298	0.49744702
Q9NR12	PDLIM7	6+7+8+9	0	-2.29356151	2.29356151	0.03463992	0.04080531	-79.6028641	0.20397136
Q9UJJ9	GNPTG	6+7+8+9	0	-1.21308435	1.21308435	0.03480921	0.04092187	-56.8654548	0.43134545
Q02747	GUCA2A	6+7+8+9	0	-0.83757711	0.83757711	0.03548979	0.04133082	-44.0417445	0.55958256
O43586	PSTPIP1	6+7+8+9	0	-1.1064874	1.1064874	0.03728143	0.04301407	-53.557659	0.46442341
P26885	FKBP2	6+7+8+9	0	-1.34354615	1.34354615	0.03745667	0.04308288	-60.5949113	0.39405089
Q9Y490	TLN1	6+7+8+9	0	-0.81732762	0.81732762	0.03745056	0.04308288	-43.2507836	0.56749216
P62328	TMSB4X	6+7+8+9	0	2.04304938	2.04304938	0.03779398	0.04342617	312.115687	4.12115687
Q96KG7	MEGF10	6+7+8+9	0	-1.16112829	1.16112829	0.0378713	0.04343293	-55.2837315	0.44716269
P33241	LSP1	6+7+8+9	0	-1.47691615	1.47691615	0.0384932	0.04382424	-64.0744078	0.35925592
P20916	MAG	6+7+8+9	0	-1.07858884	1.07858884	0.04090532	0.0459204	-52.6508262	0.47349174
P56134	ATP5MF	6+7+8+9	0	0.99962864	0.99962864	0.04136618	0.04634007	99.9485249	1.99948525
Q9GZU7	CTDSP1	6+7+8+9	0	-0.99452025	0.99452025	0.04360978	0.0480352	-49.8097253	0.50190275
Q96G27	WBP1	6+7+8+9	0	2.33531238	2.33531238	0.04575876	0.04991053	404.660223	5.04660223

Table S4: Top 25 biomarkers and two control molecules resulted from mass spectrometry screening. The upper part of the table shows the top 25 biomarkers upon ranking based on mass spectrometry results, as well as diagnostic performance (AUC and specificity), while the lower part indicates the two control molecules used in the study.

		**Mass Spectrometry Analysis**		**ROC Curve Analysis**					
	**Genes**	**Absolute Log2 Fold Change**	**q-value**	**AUC**	**Std. Error**	**95% Confidence Interval**	**p-value**	**Specificity at 90% Sensitivity**	**Specificity at 100% Sensitivity**
BIOMARKERS	B2M	1.395	2.33E-04	0.8261	0.064	0.7008 to 0.9514	0.0003	65.2	13
	VIPR1	1.452	3.06E-05	0.817	0.067	0.6861 to 0.9480	0.0006	42.9	38.1
	CALR	0.86	2.16E-07	0.8043	0.069	0.6699 to 0.9388	0.0007	47.8	34.8
	SERPINF1	1.039	9.61E-09	0.8023	0.07	0.6659 to 0.9386	0.0008	68.2	36.4
	HPX	0.952	1.58E-06	0.7761	0.07	0.6396 to 0.9125	0.0020	52.2	39.1
	IGFALS	1.1	4.09E-04	0.7761	0.072	0.6348 to 0.9174	0.0020	56.5	52.2
	KRT2	2.176	3.65E-04	0.7696	0.074	0.6250 to 0.9142	0.0025	56.5	13

	HRNR	1.912	2.91E-04	0.7522	0.076	0.6033 to 0.9010	0.0047	47.8	13
	APOA1	1.214	1.40E-05	0.7435	0.077	0.5930 to 0.8939	0.0064	52.2	17.4
	GPR180	0.998	1.28E-04	0.7432	0.079	0.5883 to 0.8980	0.0070	27.3	13.6
	KRT13	2.235	4.60E-04	0.7391	0.075	0.5913 to 0.8869	0.0074	52.2	30.4
	LRRC15	0.983	1.48E-04	0.7425	0.078	0.5902 to 0.8948	0.0087	40	20
	APOA4	0.855	1.15E-05	0.7174	0.079	0.5634 to 0.8714	0.0149	52.2	26.1
	JUP	1.848	4.76E-04	0.7185	0.08	0.5614 to 0.8757	0.0158	21.7	17.4
	ATP5F1A	2.155	2.26E-04	0.7071	0.081	0.5487 to 0.8655	0.0222	43.5	21.7
	DCD	1.915	2.13E-04	0.6978	0.082	0.5375 to 0.8581	0.0267	52.2	21.7
	CANX	1.045	1.99E-05	0.7043	0.085	0.5377 to 0.8708	0.0273	47.6	38.1
	MXRA8	0.904	1.29E-04	0.6891	0.08	0.5314 to 0.8469	0.0341	34.8	21.7
	SCGB1A1	1.412	3.30E-04	0.687	0.081	0.5273 to 0.8466	0.0363	34.8	17.4
	RNASE1	1.07	4.09E-04	0.687	0.082	0.5270 to 0.8470	0.0363	26.1	17.4
	PNP	5.845	1.10E-05	0.6739	0.082	0.5126 to 0.8352	0.0514	34.8	30.4
	CD99	1.231	1.40E-05	0.675	0.083	0.5114 to 0.8386	0.0525	36.4	31.8
	FCER2	0.834	5.78E-05	0.6717	0.084	0.5075 to 0.8360	0.0544	52.2	30.4
	VAT1	0.951	9.46E-05	0.6717	0.084	0.5077 to 0.8358	0.0544	52.2	17.4
	SCUBE3	1.485	2.03E-05	0.6746	0.086	0.5055 to 0.8438	0.0563	50	22.7
CONTROLS	CD44	0.065	0.32	0.5717	0.091	0.3936 to 0.7499	0.4217	13.0	4.3
	RNASE2	0.065	0.32	0.5326	0.090	0.3553 to 0.7099	0.7149	13.0	4.3

Table S5: Statistical analysis of the biomarkers' mass-spectrometry and ELISA quantification results. The table indicates the Minimum (Min), Maximum (Max), Mean, Median, Standard Error of the Mean (SEM) and Standard Deviation (SD) for both the mass spectrometry and the ELISA results (graphically represented as box-plots in Figure 2 and 3).

		**PEDF**		**HPX**		**CD99**		**FCER2**		**KRT13**		**HRNR**		**CANX**	
Mass Spectrometry (Figure 2)		No Tumor	Tumor	No Tumor	Tumor	No Tumor	Tumor	No Tumor	Tumor	No Tumor	Tumor	No Tumor	Tumor	No Tumor	Tumor
	Min	20587.0	20875.5	3759929.8	2856963.3	104718.1	74844.9	29750.7	34719.7	293610.6	184933.7	7521.8	3852.5	7840.5	12820.1
	Max	223883.1	91576.5	25157592.0	9157538.0	3106647.0	1400008.5	986700.9	388217.0	39971668.0	3095748.8	654237.6	99452.6	167766.2	79652.4
	Mean	93196.7	45345.5	10687672.6	5522924.9	1057592.1	450481.5	311839.3	174952.7	4303372.0	914048.0	78622.9	20893.1	70107.4	33976.0
	Median	80240.9	43947.5	8246233.0	5110637.8	620543.2	323484.1	277933.0	142632.9	1996926.8	597343.1	28265.2	14847.2	47928.3	30632.6
	SEM	10836.5	4553.8	1413418.9	428909.6	207774.4	78865.1	49249.3	19977.9	1781308.1	167487.6	29534.0	4577.7	10702.0	3718.1
	SD	51970.0	20365.0	6778519.0	1918142.2	996451.1	352695.6	236191.6	89344.1	8542853.6	749027.4	141640.3	20471.9	51324.8	16627.7
ELISA Validation (Figure 3A)		No Tumor	Tumor	No Tumor	Tumor	No Tumor	Tumor	No Tumor	Tumor	No Tumor	Tumor	No Tumor	Tumor	No Tumor	Tumor
	Min	0.002	0.000	0.038	0.007	0.002	0.001	1.175	0.335	0.033	0.003	0.000	0.000	0.002	0.000
	Max	0.547	0.037	2.228	0.375	0.342	0.022	409.079	58.690	1.006	0.276	1418.180	171.853	0.217	0.054
	Mean	0.057	0.009	0.569	0.110	0.049	0.009	47.447	9.005	0.327	0.086	154.747	22.338	0.049	0.012
	Median	0.012	0.004	0.144	0.082	0.022	0.004	10.975	3.907	0.192	0.033	25.364	5.052	0.018	0.004
	SEM	0.025	0.002	0.143	0.025	0.018	0.002	20.672	3.009	0.063	0.021	71.016	8.712	0.014	0.004
	SD	0.118	0.011	0.684	0.113	0.084	0.008	99.140	13.458	0.303	0.095	340.581	38.961	0.066	0.016
ELISA Validation (Figure 3B)		Low grade	High-grade	Low grade	High-grade	Low grade	High-grade	Low grade	High-grade	Low grade	High-grade	Low grade	High-grade	Low grade	High-grade
	Min	0.001	0.000	0.031	0.007	0.001	0.001	0.818	0.335	0.018	0.003	0.000	0.000	0.000	0.000
	Max	0.547	0.033	2.228	0.375	0.342	0.022	409.079	58.690	1.006	0.276	1418.180	171.853	0.217	0.054
	Mean	0.051	0.008	0.506	0.101	0.043	0.009	41.782	8.956	0.292	0.085	134.851	22.809	0.043	0.012
	Median	0.012	0.004	0.137	0.082	0.015	0.004	10.893	3.907	0.180	0.033	25.033	5.052	0.014	0.004
	SEM	0.021	0.002	0.125	0.026	0.015	0.002	17.767	3.574	0.057	0.024	61.033	10.642	0.012	0.004
	SD	0.110	0.010	0.650	0.104	0.079	0.008	92.319	14.296	0.294	0.095	317.138	42.569	0.063	0.017

Table S6: Immunohistochemical staining of eleven prostate adenocarcinoma cases. Expression of biomarkers in malignant and benign gland areas were evaluated with a four-tired score (0= negative, 1=weak, 2=moderate, 3=strong) and the extend of stained areas was estimated in 10% increments.

Biomarker	Patient	GS	Benign intensity	Benign percentage	Staining cell type (0=luminal, 1=basal, 2=both)	Note	Malignant intensity	Malignant percentage	Note
PEDF	1	7a	1	10	2	cytoplasm	0	100	
	2	7a	1	70	0	cytoplasm	1	10	cytoplasm
	3	7b	1	< 10%	2	cytoplasm	1	40	cytoplasm/nucleus
	4	7b	1	40	2	cytoplasm/membrane	1	40	cytoplasm
	5	7b	1	100	2	cytoplasm	1	40	cytoplasm
	6	7b	1	80	2	cytoplasm	1	60	cytoplasm
	7	8	2	< 10%	1	cytoplasm	1	30	cytoplasm
	8	9a	1	50	2	cytoplasm	1	90	cytoplasm
	9	9a	1	< 10%	0	cytoplasm	1	20	cytoplasm
	10	9a	1	30	2	cytoplasm	1	10	cytoplasm
	11	9a	1	80	2	cytoplasm	1	30	cytoplasm
HPX	1	7a	1	100	2	cytoplasm/membrane	1	100	cytoplasm/membrane
	2	7a	3	100	2	cytoplasm/membrane/nucleus	2	100	cytoplasm/membrane
	3	7b	2	100	2	cytoplasm/membrane	2	100	cytoplasm/membrane
	4	7b	1	100	2	cytoplasm/membrane/nucleus	1	100	cytoplasm
	5	7b	3	90	2	cytoplasm/membrane/nucleus	3	100	cytoplasm/membrane
	6	7b	1	100	2	cytoplasm/membrane/nucleus	1	100	cytoplasm/membrane
	7	8	2	100	2	cytoplasm/membrane	1	100	cytoplasm/membrane
	8	9a	1	100	2	cytoplasm/membrane/nucleus	2	100	cytoplasm/membrane
	9	9a	2	60	2	cytoplasm/membrane/nucleus	1	100	cytoplasm
	10	9a	1	100	2	cytoplasm/membrane/nucleus	1	100	cytoplasm/membrane
	11	9a	1	20	2	cytoplasm/membrane/nucleus	1	100	cytoplasm/membrane
CD99	1	7a	2	80	2	dot like/membrane	1	90	dot like/membrane
	2	7a	2	70	2	dot like/membrane	2	90	dot like
	3	7b	1	80	2	dot like/membrane	2	70	dot like
	4	7b	1	90	2	dot like	2	20	dot like/membrane
	5	7b	2	90	2	membrane	1	70	dot like
	6	7b	2	60	0	dot like/membrane	1	40	dot like
	7	8	2	80	0	dot like	2	70	dot like
	8	9a	2	90	2	dot like	1	80	dot like/membrane
	9	9a	2	60	0	dot like	1	30	dot like
	10	9a	2	80	0	dot like	2	80	dot like
	11	9a	1	40	0	dot like	2	80	dot like/membrane
CANX	1	7a	3	100	2	cytoplasm/membrane	3	100	cytoplasm/membrane
	2	7a	3	100	2	cytoplasm	3	100	cytoplasm
	3	7b	3	100	2	cytoplasm/membrane	2	100	cytoplasm/membrane
	4	7b	3	100	2	cytoplasm/membrane	2	100	cytoplasm
	5	7b	3	100	2	cytoplasm	3	100	cytoplasm

	6	7b	3	100	2	cytoplasm/membrane	2	100	cytoplasm
	7	8	2	100	2	cytoplasm/membrane	2	100	cytoplasm/membrane
	8	9a	3	100	2	cytoplasm	2	100	cytoplasm/membrane
	9	9a	3	100	2	cytoplasm	2	100	cytoplasma
	10	9a	2	100	2	cytoplasm/membrane	3	90	cytoplasm
	11	9a	2	100	2	cytoplasm	2	100	cytoplasm/membrane
**FCER2**	1	7a	0	100			0	100	
	2	7a	0	100			0	100	
	3	7b	0	100			0	100	
	4	7b	0	100			0	100	
	5	7b	0	100			0	100	
	6	7b	0	100			0	100	
	7	8	0	100			0	100	
	8	9a	0	100			0	100	
	9	9a	0	100			0	100	
	10	9a	0	100			0	100	
	11	9a	0	100			0	100	
**HRNR**	1	7a	1	80	2	cytoplasm	1	100	cytoplasm
	2	7a	1	40	0	dot like/cytoplasm	3	100	dot like/cytoplasm
	3	7b	1	60	2	cytoplasm/dot like	1	50	dot like/cytoplasm
	4	7b	1	10	0	dot like	1	100	dot like/cytoplasm
	5	7b	1	50	2	dot like/cytoplasm	2	90	cytoplasm
	6	7b	1	90	0	dot like/cytoplasm	1	50	dot like/cytoplasm
	7	8	1	90	2	cytoplasm/dot like	1	70	cytoplasm
	8	9a	1	90	2	cytoplasm/dot like	1	100	dot like/cytoplasm
	9	9a	1	100	2	cytoplasm/dot like	1	100	cytoplasm
	10	9a	1	10	2	cytoplasm/dot like	1	20	cytoplasm
	11	9a	1	100	2	cytoplasm	1	100	cytoplasm/dot like
**KRT13**	1	7a	2	40	1	staining mainly peripheral zone	0	100	
	2	7a	3	40	1		0	100	
	3	7b	2	30	1		0	100	
	4	7b	2	30	1		0	100	
	5	7b	2	20	1		0	100	
	6	7b	2	10	1		0	100	
	7	8	2	40	1		0	100	
	8	9a	2	20	1		0	100	
	9	9a	3	70	1		0	100	
	10	9a	2	20	1		0	100	
	11	9a	2	70	1		0	100	
